# Supplementary material for: Long-read and chromosome-scale assembly of the hexaploid wheat genome achieves high resolution for research and breeding
Source: Gigascience. 2022 Apr 28;11:giac034. doi: 10.1093/gigascience/giac034 (PMC9049114; doi:10.1093/gigascience/giac034)

Table S1: Statistics of the ONT sequencing data

	Raw ONT reads	Longest reads
Number of runs	2 MinION + 18 PromethION	
Cumulative size	1,074 Gb	510 Gb
# of reads	120,883,721	11,654,667
Coverage (17Gb)	63 X	30 X
N50 (bp)	24,651	45,077
Coverage (>50Kb)	14.1 X	14.1 X

Table S2: BNG Optical map

	DLE-1 genome map
Genome map number	1,053
Total Genome Map Length (Mbp)	14,947
Genome Map N50 (Mbp)	37.5

Table S3: Raw long-read assemblies

	SMARTDeNovo	Redbean	Flye
Subset of reads used	Longest - 30X	Longest - 30X	Longest - 30X
Cumulative size	**14,155,561,280**	29,695,324,870	13,050,586,822
# contigs	21,197	646,779	**14,205**
longest contig (bp)	13,367,134	957,564	**18,693,219**
minimal size (bp)	34,696	1,157	50,033
N50 (L50)	1,142,720 (3,758)	76,231 (107,967)	**1,775,593 (2,165)**
N90 (L90)	322,974 (12,488)	21,181 (397,125)	**471,502 (7,566)**
Restitution time (h)	408	168	1,032
IT	8 nodes with 32-core and 1.5Tb	1 node of 64-core and 3Tb	1 node of 64-core and 3Tb

Table S4: Hybrid assemblies obtained using ONT and BNG data

		SMARTDeNovo	Flye
Scaffolds	Cumulative size	**14,272,571,061**	14,204,637,528
	# scaffolds	4,255	**4,135**
	longest scaffold (bp)	**253,607,211**	252,437,931
	N50 (L50)	48,460,876 (79)	**49,688,204 (77)**
	N90 (L90)	7,877,958 (378)	**8,121,250 (362)**
	% of unknown bases	**1.81%**	5.99%
Contigs	Cumulative size	**14,014,403,266**	13,330,835,323
	# contigs	**14,241**	15,282
	longest contig (bp)	15,116,800	**19,215,531**
	N50 (L50)	**2,144,699 (1,963)**	1,662,574 (2,131)
	N90 (L90)	**595,732 (6,672)**	440,639 (8,204)
	BUSCO<br>(n=4,896)	**Complete : 97.0%**<br>**Duplicated : 87.3%**<br>**Fragmented : 0.4%**<br>**Missing : 2.6%**	Complete : 96.7%<br>Duplicated : 86.6%<br>**Fragmented : 0.4%**<br>Missing : 2.9%

Table S5: Impact of the polishing

		SMARTDeNovo raw	SMARTDeNovo polished with long reads	SMARTDeNovo polished with long and short reads
BUSCO (n=4,896)	Complete	83.0%	96.7%	96.6%
	Duplicated	32.5%	83.1%	87.0%
	Fragmented	2.4%	0.6%	0.6%
	Missing	14.6%	2.7%	2.8%
IBSPs (n=5.76 M)	Aligned	80.4%	92.9%	93.4%
	Perfectly aligned	7.0%	28.0%	58.9%

Table S6: Statistics of the ONT reads obtained with two different versions of the guppy basecaller

	Guppy 3.3	Guppy 3.6
Number of runs	2 MinION + 18 PromethION<br>Selection of the longest reads (30X)	
Cumulative size	510 Gb	510 Gb
# of reads	11,731,678	11,654,667
Coverage (17Gb)	30 X	30 X
N50<br>(bp)	44,797	45,077
Coverage (>50Kb)	13.9 X	14.1 X

Table S7: Long-read assemblies with ONT reads obtained with two different versions of the guppy basecaller

	SMARTDeNovo	SMARTDeNovo
Subset of reads used	Guppy 3.3<br>Longest - 30X	Guppy 3.6<br>Longest - 30X
Cumulative size	13,975,909,499	**14,155,561,280**
# contigs	26,167	**21,197**
longest contig (bp)	6,593,358	**13,367,134**
minimal size (bp)	33,360	**34,696**
N50 (L50)	899,898 (4,685)	**1,142,720 (3,758)**
N90 (L90)	247,425 (15,801)	**322,974 (12,488)**

Table S8: RNASeq data for Renan from 28 samples corresponding to 14 different organs/conditions in replicates.

Samples accession	Genotype	Zadoks condition	Type	Tissue	Wheat Ontology (CO)	Plant Ontology (PO)	Stress Ontology (PSO)
SAMEA11349165	Renan	100DD	control	Grain	CO_321:0001679	PO:0030104	
SAMEA11349166	Renan	500DD	control	Grain	CO_321:0001679	PO:0030104	
SAMEA11349167	Renan	250DD	control	Grain	CO_321:0001679	PO:0030104	
SAMEA11349168	Renan	500DD	control	Grain	CO_321:0001679	PO:0030104	
SAMEA11349169	Renan	250DD	control	Grain	CO_321:0001679	PO:0030104	
SAMEA11349170	Renan	100DD	stress	Grain	CO_321:0001679	PO:0030104	PSO:0000012
SAMEA11349171	Renan	500DD	stress	Grain	CO_321:0001679	PO:0030104	PSO:0000012
SAMEA11349172	Renan	100DD	stress	Grain	CO_321:0001679	PO:0030104	PSO:0000012
SAMEA11349173	Renan	100DD	control	Grain	CO_321:0001679	PO:0030104	
SAMEA11349174	Renan	250DD	stress	Grain	CO_321:0001679	PO:0030104	PSO:0000012
SAMEA11349175	Renan	500DD	stress	Grain	CO_321:0001679	PO:0030104	PSO:0000012
SAMEA11349176	Renan	250DD	stress	Grain	CO_321:0001679	PO:0030104	PSO:0000012
SAMEA11349177	Renan	700DD	control	Grain	CO_321:0001679	PO:0030104	
SAMEA11349178	Renan	700DD	control	Grain	CO_321:0001679	PO:0030104	
SAMEA11349179	Renan	700DD	stress	Grain	CO_321:0001679	PO:0030104	PSO:0000012
SAMEA11349180	Renan	700DD	stress	Grain	CO_321:0001679	PO:0030104	PSO:0000012
SAMEA11349181	Renan	Z13	control	Root	CO_321:0000476/10	PO:0009005	
SAMEA11349182	Renan	Z13	control	Root	CO_321:0000476/10	PO:0009005	
SAMEA11349183	Renan	Z13	control	Leaves	CO_321:0000476/29	PO:0025034	
SAMEA11349184	Renan	Z13	control	Leaves	CO_321:0000476/29	PO:0025034	
SAMEA11349185	Renan	Z32	control	Leaves	CO_321:0000476/29	PO:0025034	
SAMEA11349186	Renan	Z32	control	Leaves	CO_321:0000476/29	PO:0025034	
SAMEA11349187	Renan	Z61	control	Leaves	CO_321:0000476/29	PO:0025034	
SAMEA11349188	Renan	Z61	control	Leaves	CO_321:0000476/29	PO:002503	
SAMEA11349189	Renan	Z61	control	Stem	CO_321:0000476/46	PO:0009047	
SAMEA11349190	Renan	Z61	control	Stem	CO_321:0000476/46	PO:0009047	
SAMEA11349191	Renan	Z32	control	Stem	CO_321:0000476/46	PO:0009047	
SAMEA11349192	Renan	Z61	control	Stem	CO_321:0000476/46	PO:0009047	

Figure S1. Validation of both ends, which contains telomeric repeats, of the chromosome 7A The optical maps are in blue and the chromosome sequence in green. Restriction sites are represented by vertical lines and are joined between the sequence and the map when properly aligned..

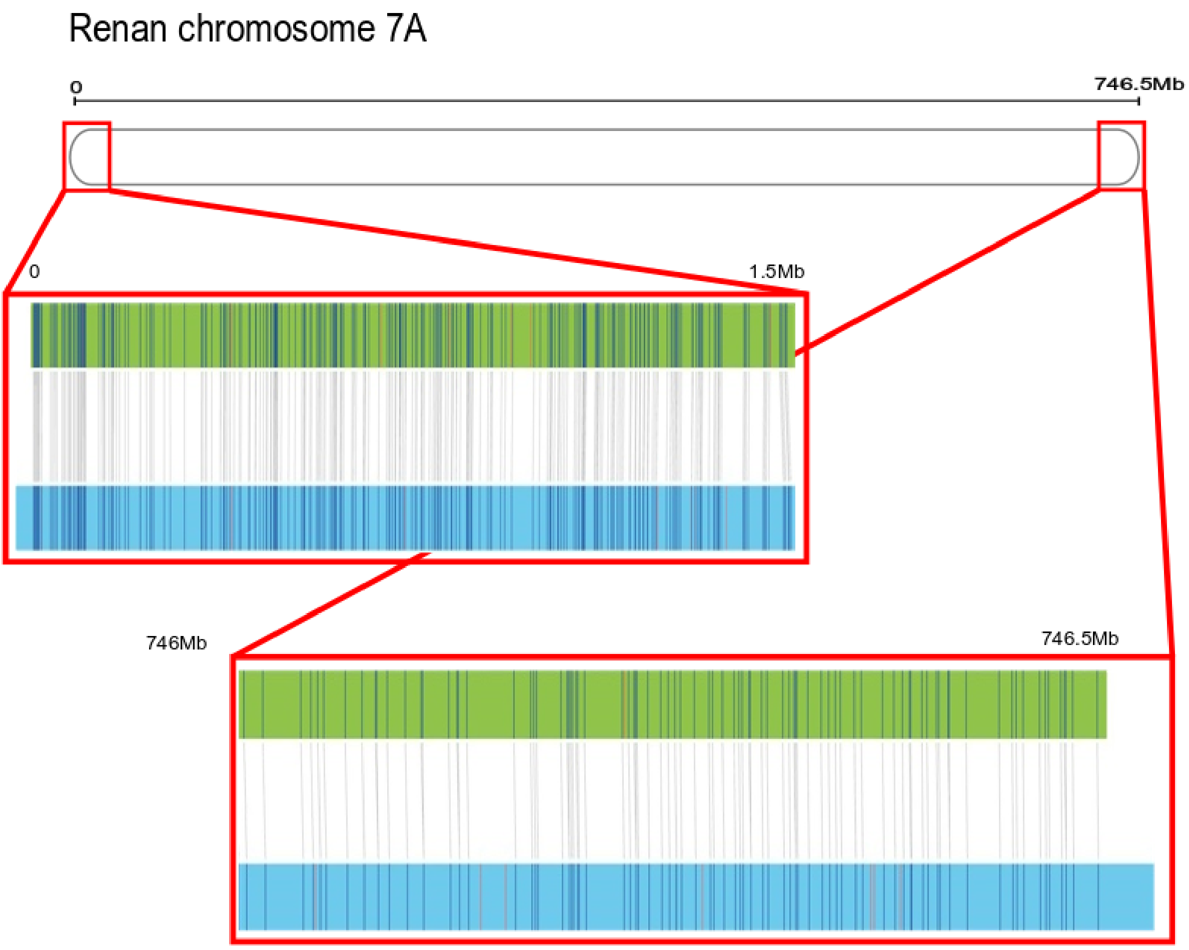

Figure S2. Comparison of the accuracy of different ONT basecallers. **A.** ONT reads from a yeast sample. **B.** ONT ultra-long reads (>100 kb) from a wheat sample.

A.

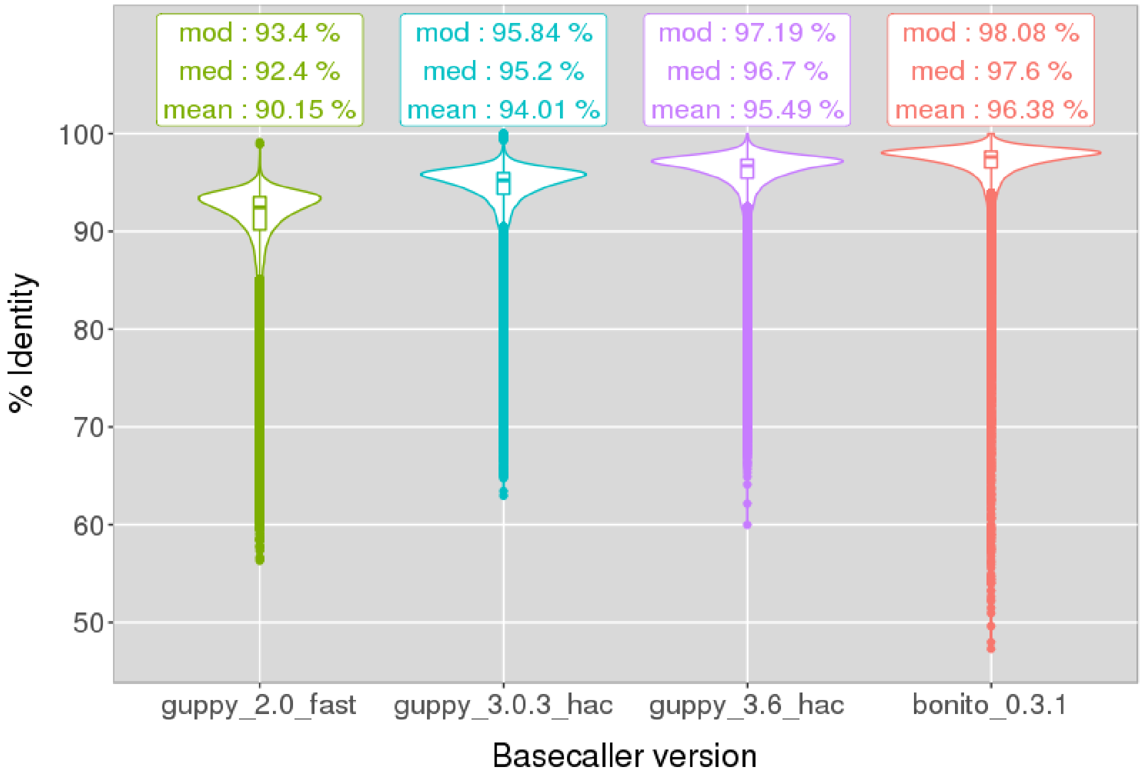

B.

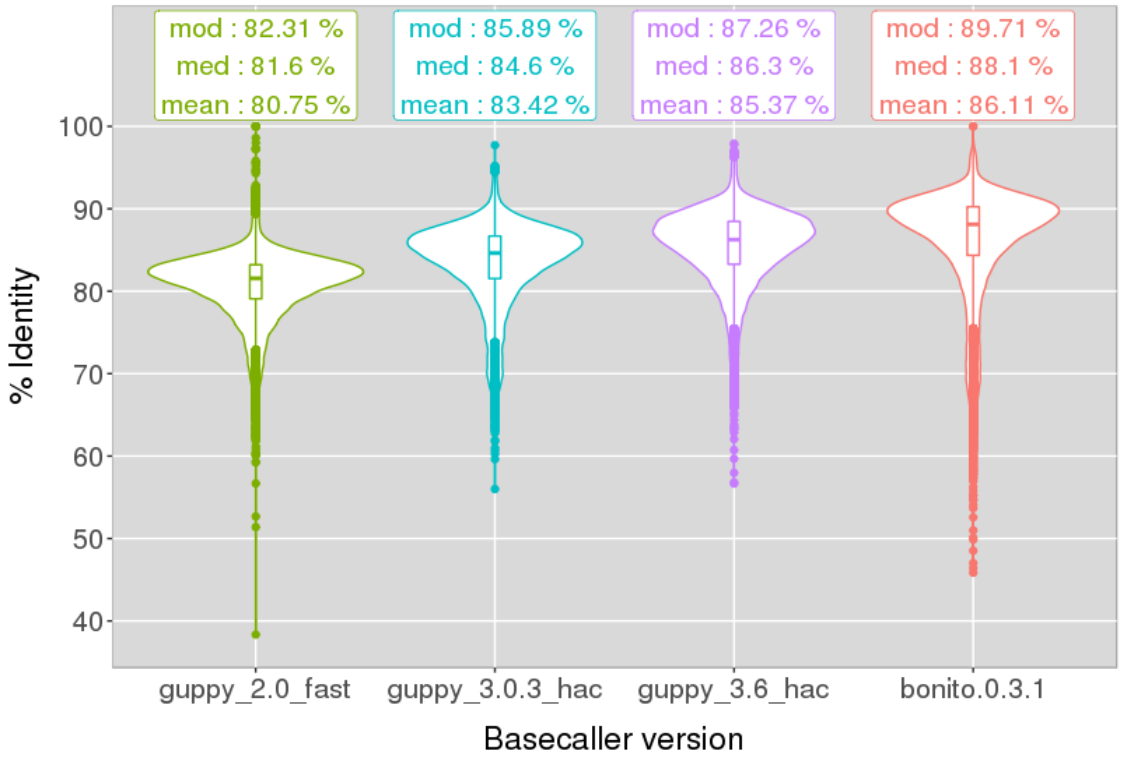

Figure S3. Number of gaps per Mbp in Chinese Spring and Renan genome assemblies.

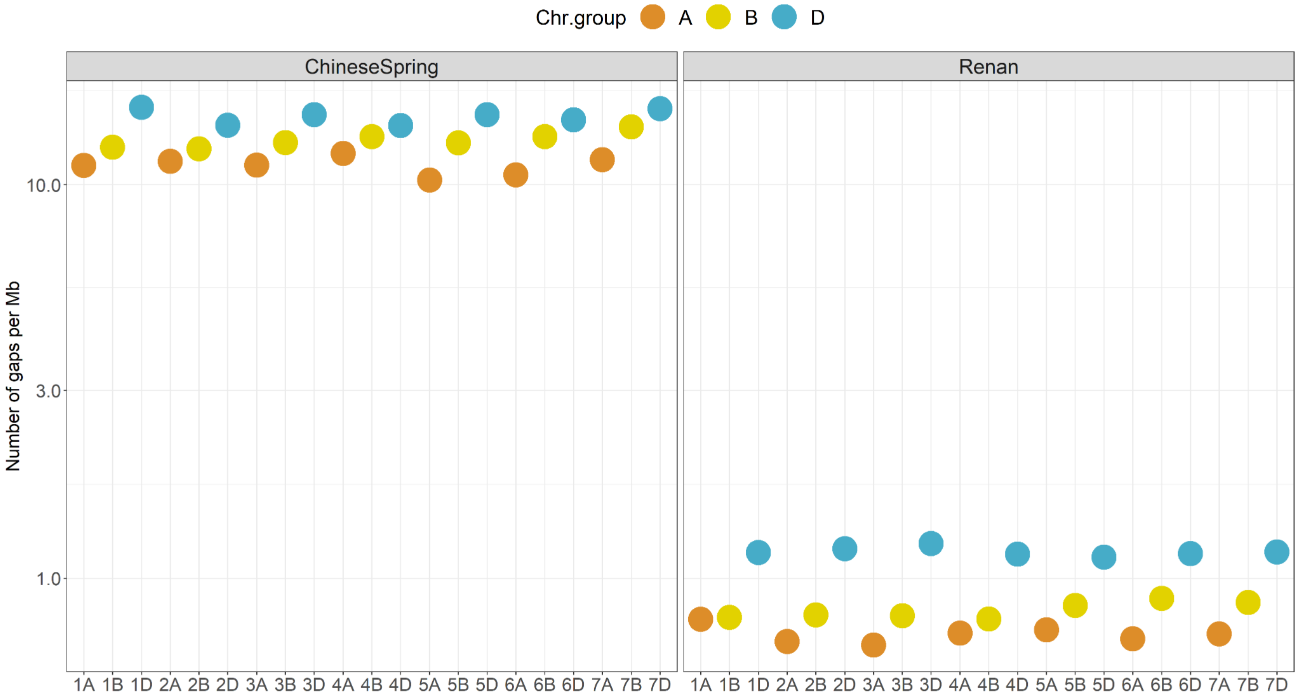

Figure S4. Large-scale structural variation in chromosomes 5B and 7B of ArinaLrFor and SY Mattis cultivars. Translocated regions correspond to the blocks in cyan (chromosome 5B) and green (chromosome 7B) that appear specific to SY Mattis and ArinaLrFor.

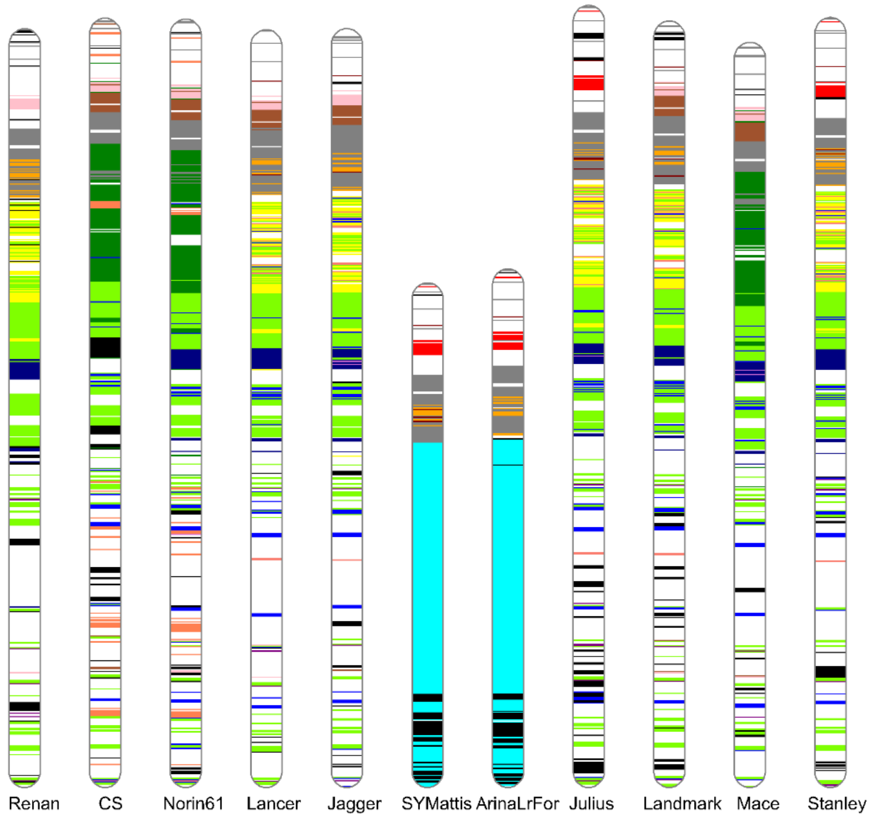

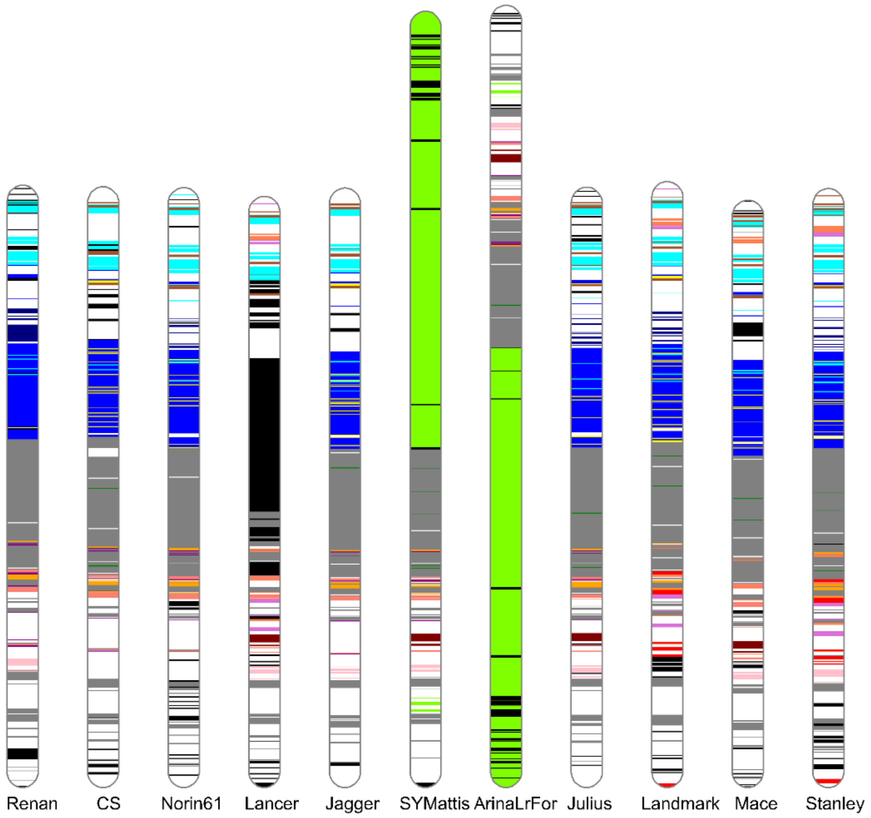

Figure S5. Validation of the omega gliadin gene cluster using Nanopore long-reads. Violet rectangles represent reads longer than 50Kb, yellow boxes represent the omega genes and the red box represent a sequence gap.

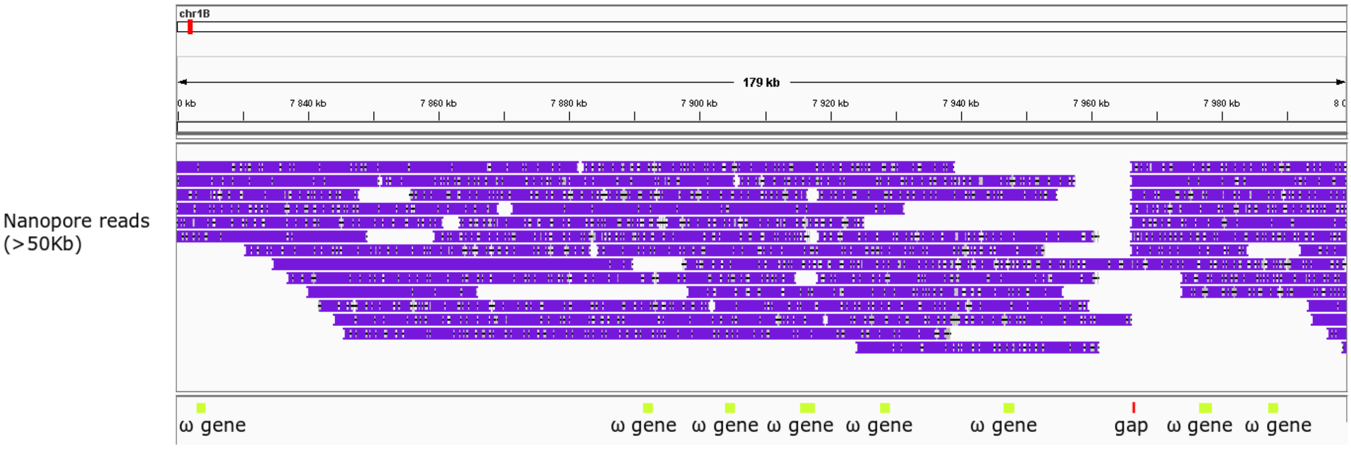

Supplement: giac034_Supplemental_Files [file giac034_supplemental_files.zip › Supplementary Data.pdf]
