# Supplementary material for: Long-read and chromosome-scale assembly of the hexaploid wheat genome achieves high resolution for research and breeding
Source: Gigascience. 2022 Apr 28;11:giac034. doi: 10.1093/gigascience/giac034 (PMC9049114; doi:10.1093/gigascience/giac034)
Supplement: giac034_Supplemental_Files [file giac034_supplemental_files.zip › Supplementary File.pdf]

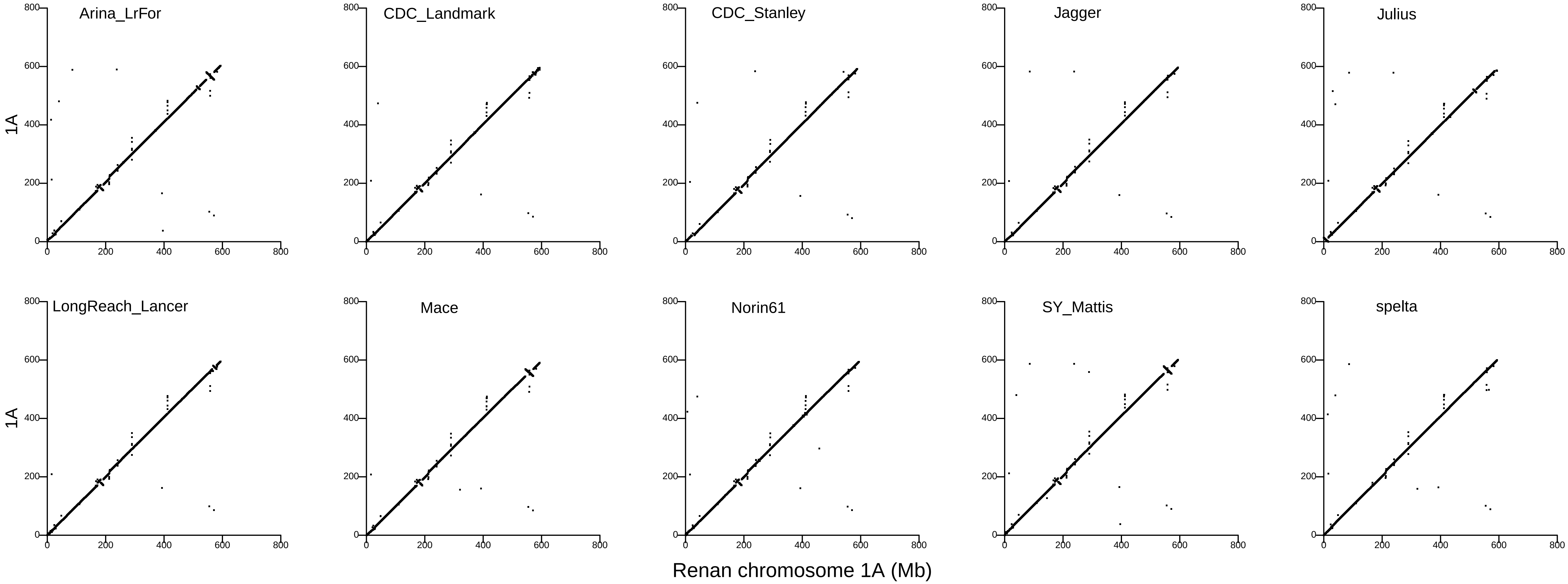

Arina_LrFor
CDC_Landmark
CDC_Stanley
Jagger
Julius
LongReach_Lancer
Mace
Norin61
SY_Mattis
spelta
1A
0
200
400
600
800
Renan chromosome 1A (Mb)

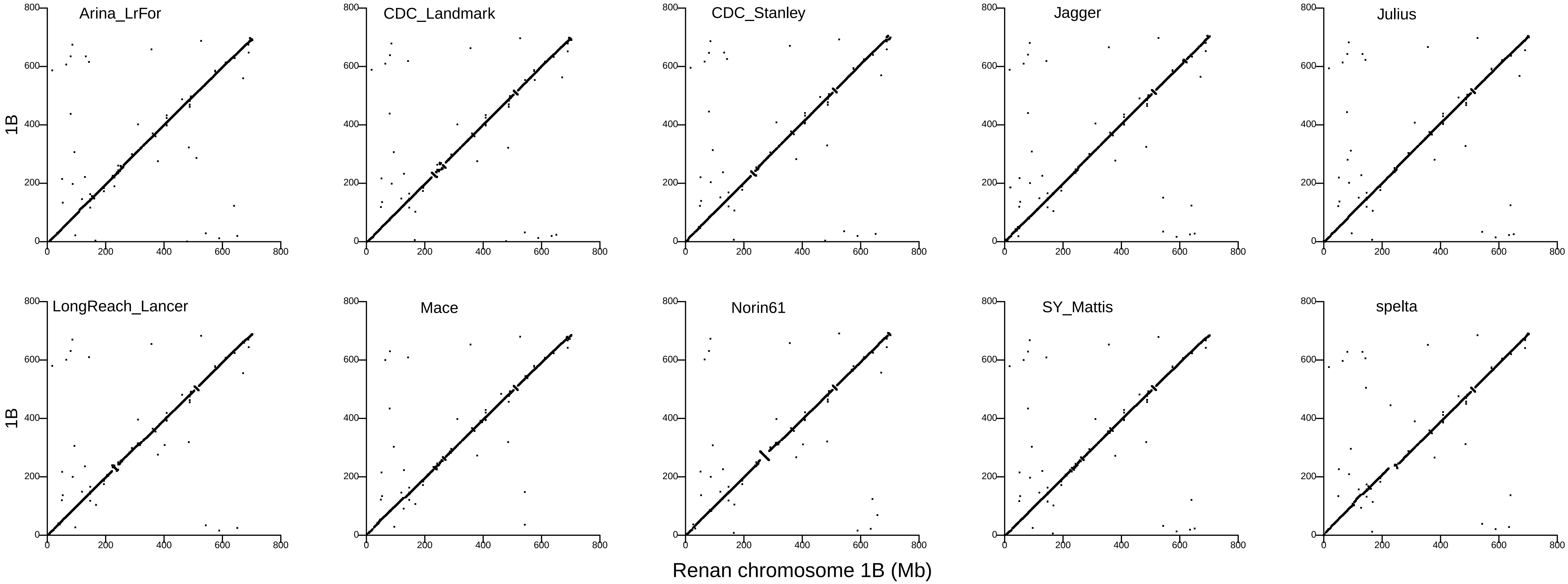
Arina_LrFor
CDC_Landmark
CDC_Stanley
Jagger
Julius
LongReach_Lancer
Mace
Norin61
SY_Mattis
spelta
1B
1B
0
200
400
600
800
Renan chromosome 1B (Mb)

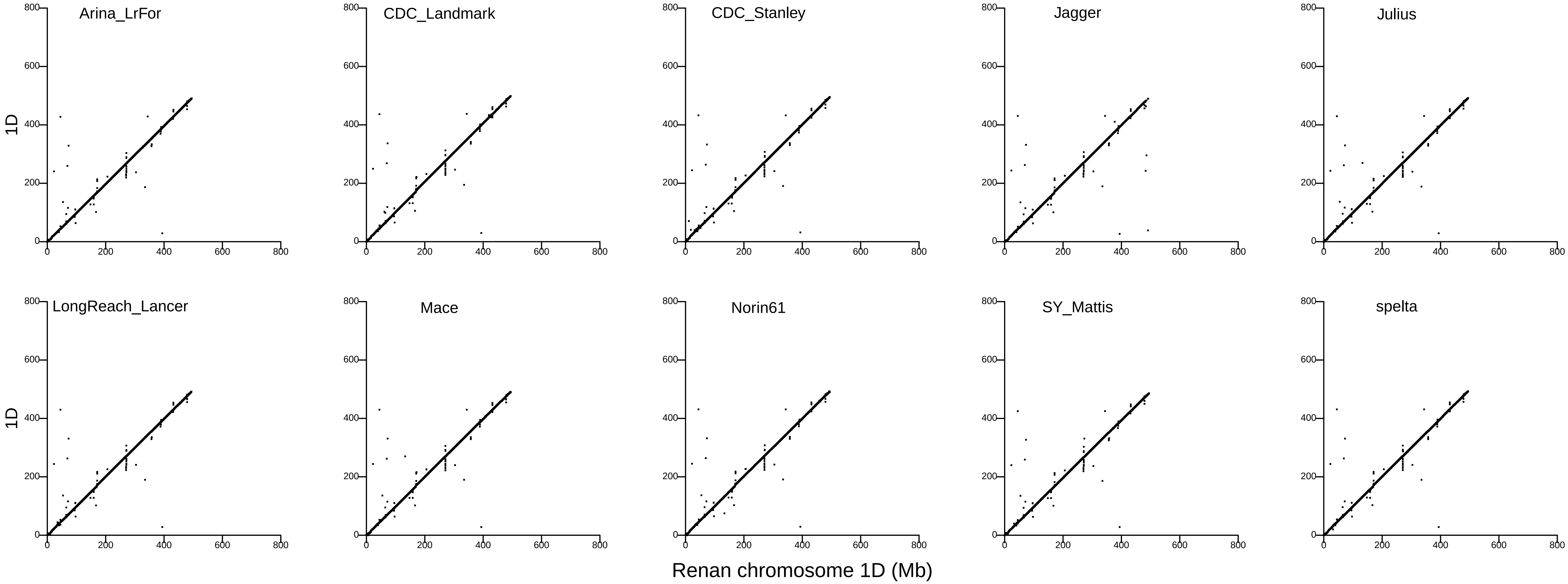
Arina_LrFor
CDC_Landmark
CDC_Stanley
Jagger
Julius
LongReach_Lancer
Mace
Norin61
SY_Mattis
spelta
1D
0
200
400
600
800
Renan chromosome 1D (Mb)

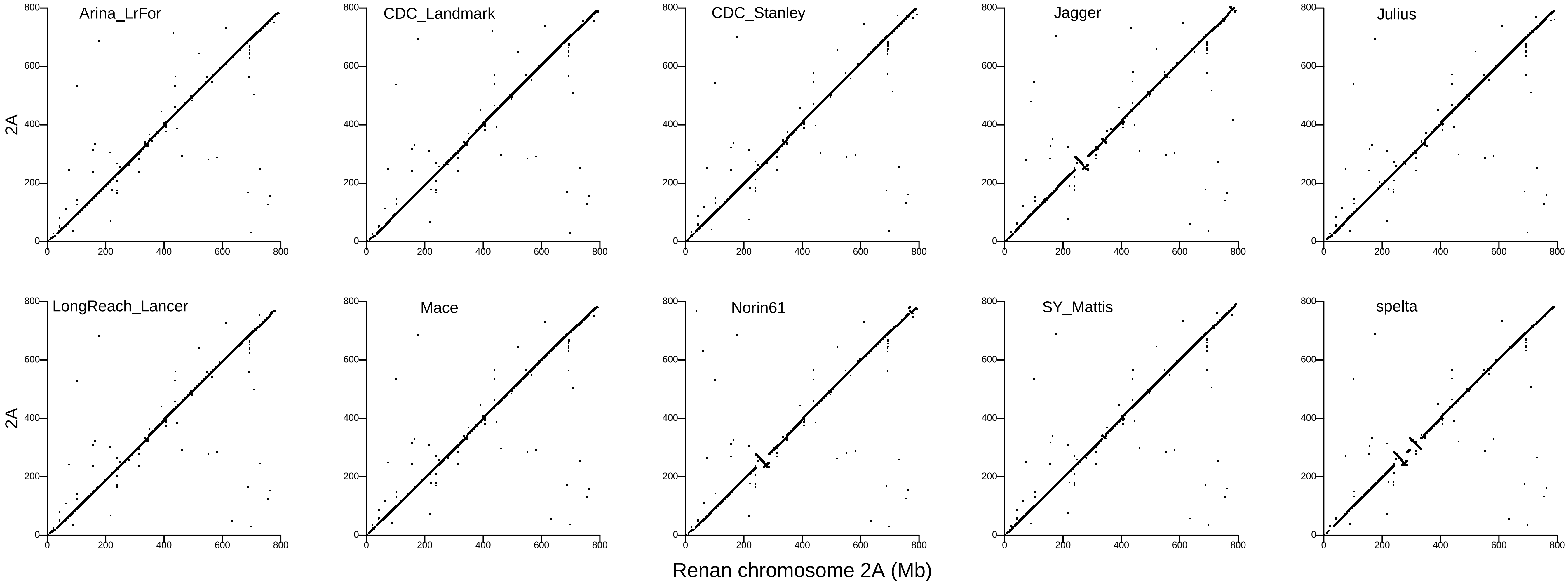

Arina_LrFor
CDC_Landmark
CDC_Stanley
Jagger
Julius
LongReach_Lancer
Mace
Norin61
SY_Mattis
spelta
2A
2A
0
200
400
600
800
Renan chromosome 2A (Mb)

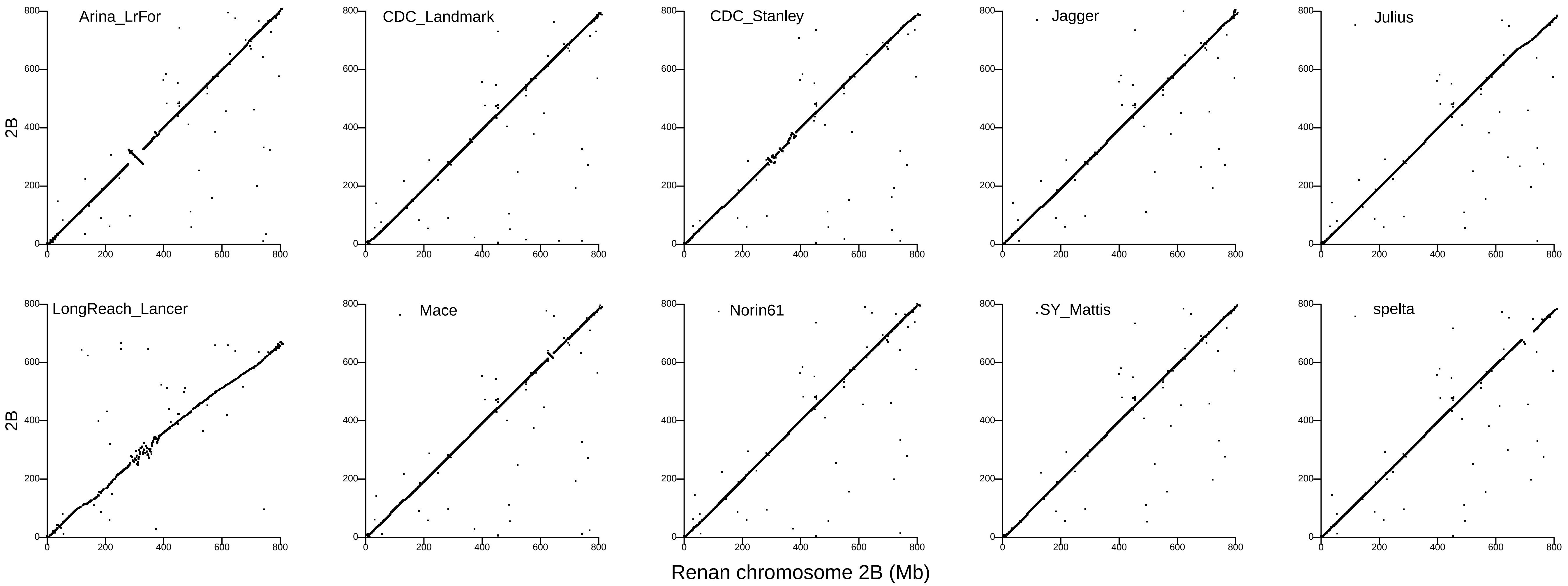

Arina_LrFor
CDC_Landmark
CDC_Stanley
Jagger
Julius
LongReach_Lancer
Mace
Norin61
SY_Mattis
spelta
2B
0
200
400
600
800
Renan chromosome 2B (Mb)

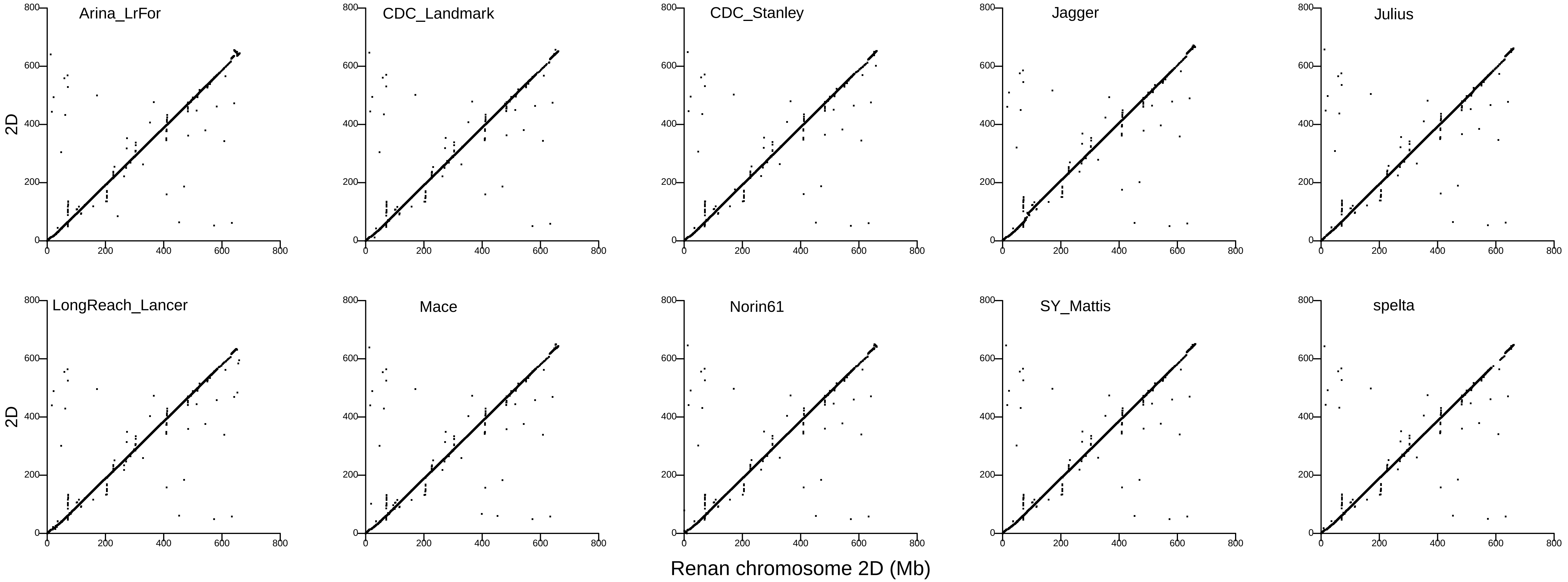
Arina_LrFor
CDC_Landmark
CDC_Stanley
Jagger
Julius
LongReach_Lancer
Mace
Norin61
SY_Mattis
spelta
2D
Renan chromosome 2D (Mb)
0
200
400
600
800

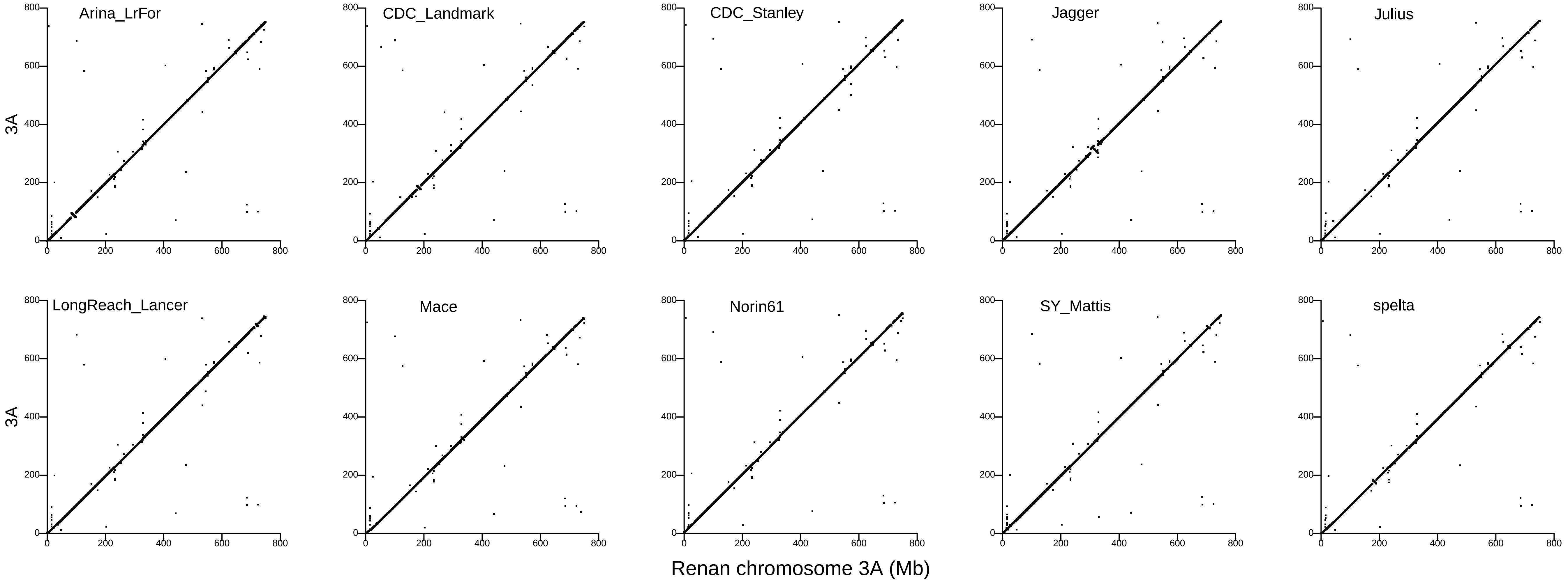

Arina_LrFor
CDC_Landmark
CDC_Stanley
Jagger
Julius
LongReach_Lancer
Mace
Norin61
SY_Mattis
spelta
3A
3A
0
200
400
600
800
Renan chromosome 3A (Mb)

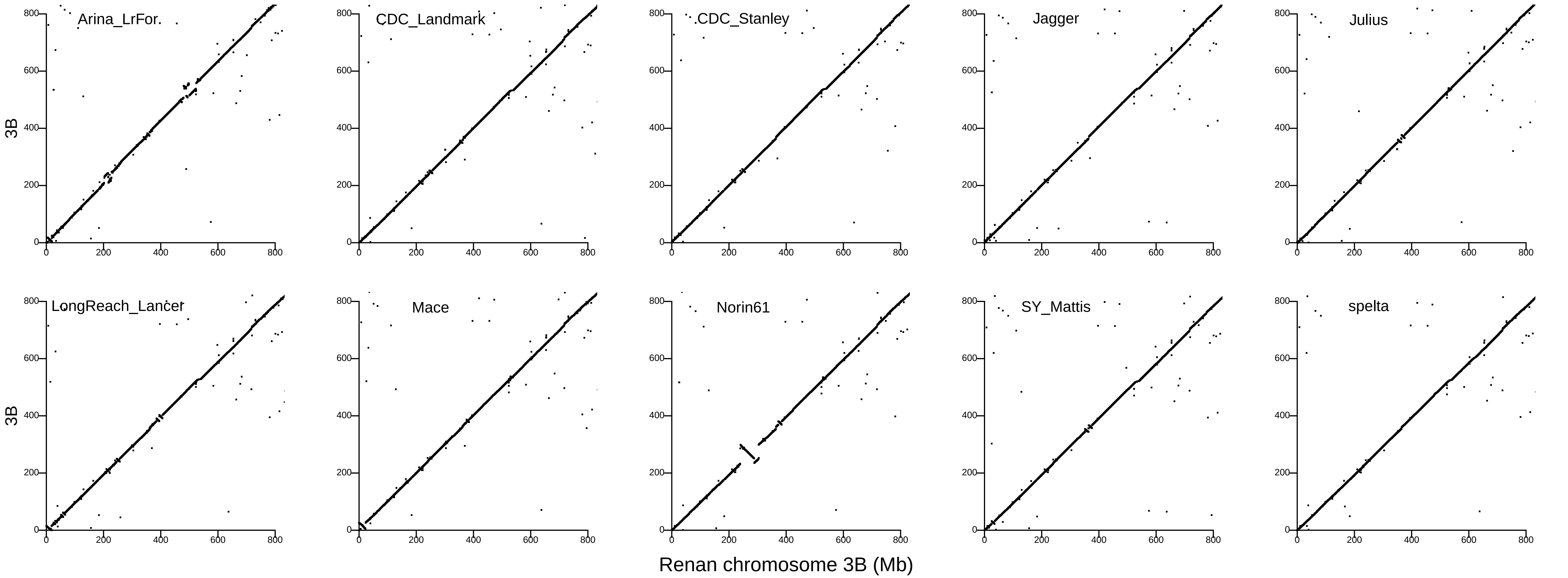

Arina_LrFor.
CDC_Landmark
CDC_Stanley
Jagger
Julius
LongReach_Lancer
Mace
Norin61
SY_Mattis
spelta
3B
Renan chromosome 3B (Mb)

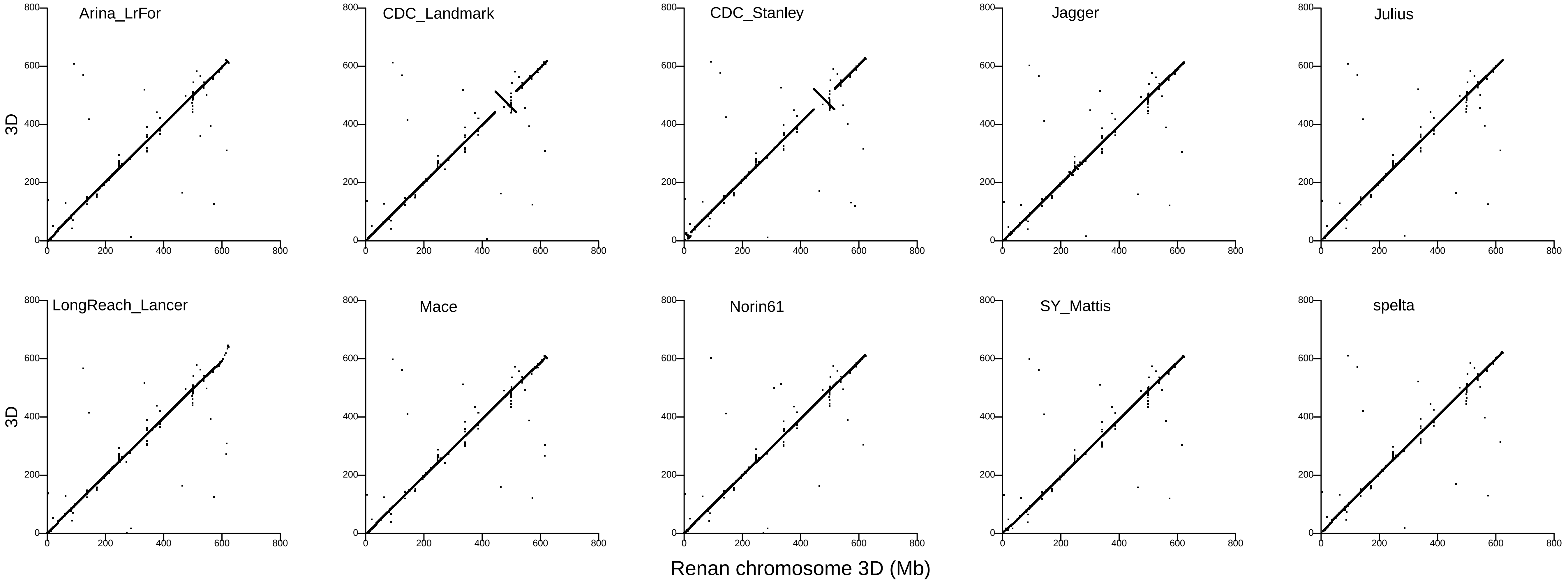

Arina_LrFor
CDC_Landmark
CDC_Stanley
Jagger
Julius
LongReach_Lancer
Mace
Norin61
SY_Mattis
spelta
3D
0
200
400
600
800
Renan chromosome 3D (Mb)

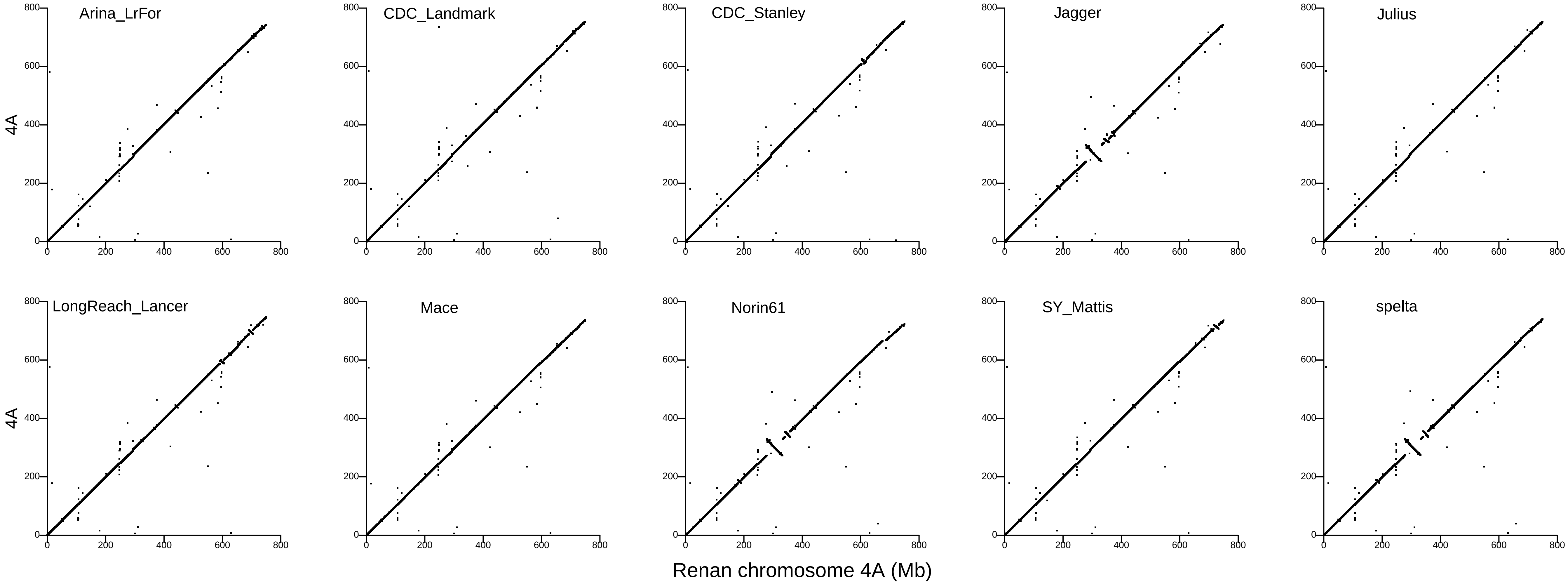

Arina_LrFor
CDC_Landmark
CDC_Stanley
Jagger
Julius
LongReach_Lancer
Mace
Norin61
SY_Mattis
spelta
4A
Renan chromosome 4A (Mb)

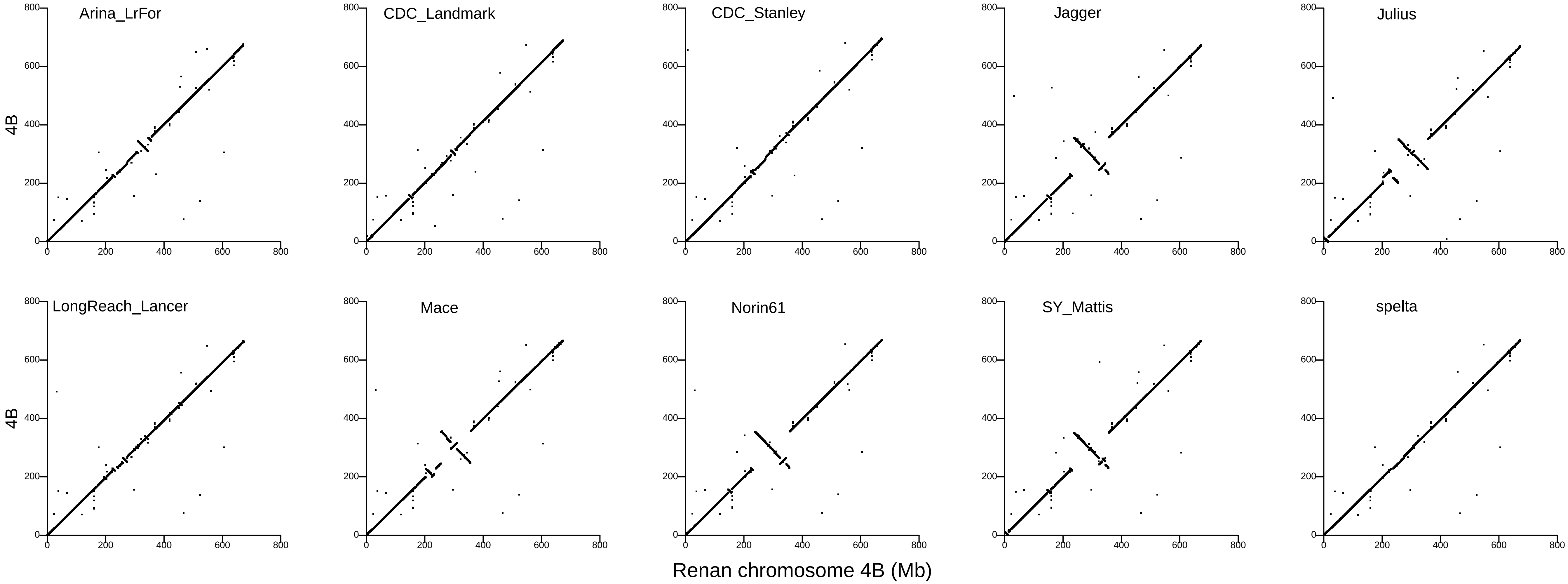

Arina_LrFor
CDC_Landmark
CDC_Stanley
Jagger
Julius
LongReach_Lancer
Mace
Norin61
SY_Mattis
spelta
4B
4B
0
200
400
600
800
Renan chromosome 4B (Mb)

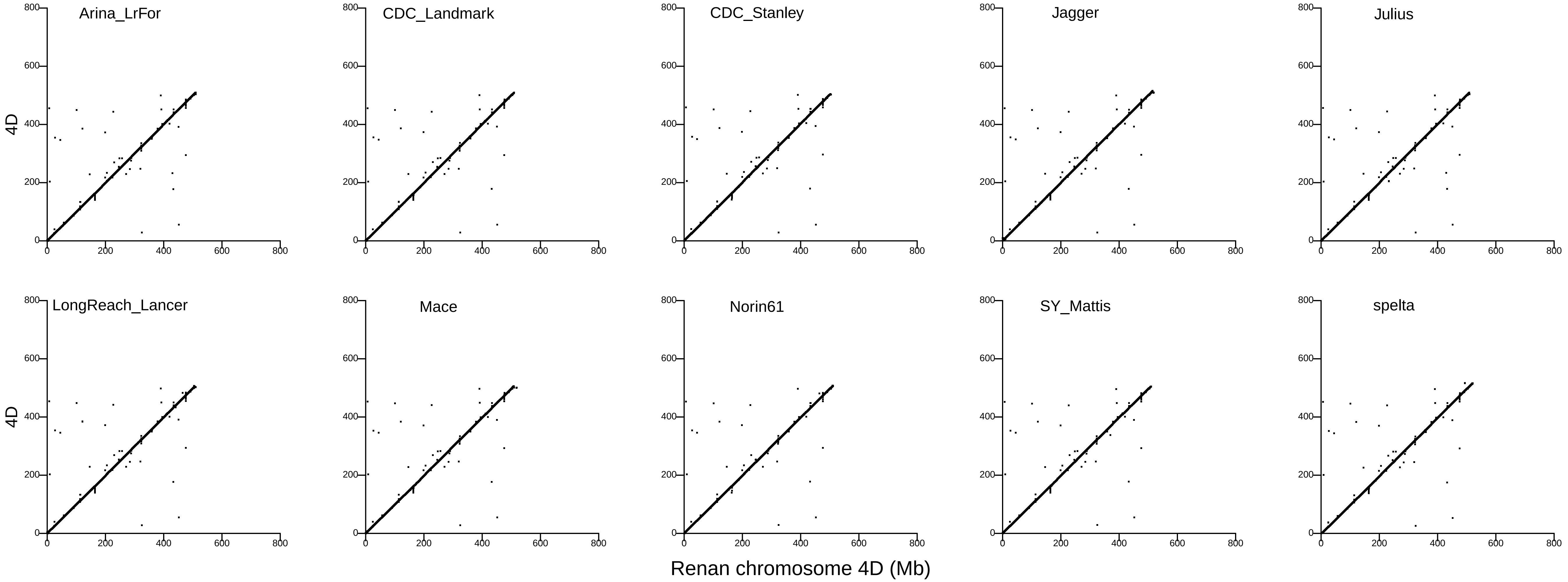

Arina_LrFor
CDC_Landmark
CDC_Stanley
Jagger
Julius
LongReach_Lancer
Mace
Norin61
SY_Mattis
spelta
4D
0
200
400
600
800
Renan chromosome 4D (Mb)

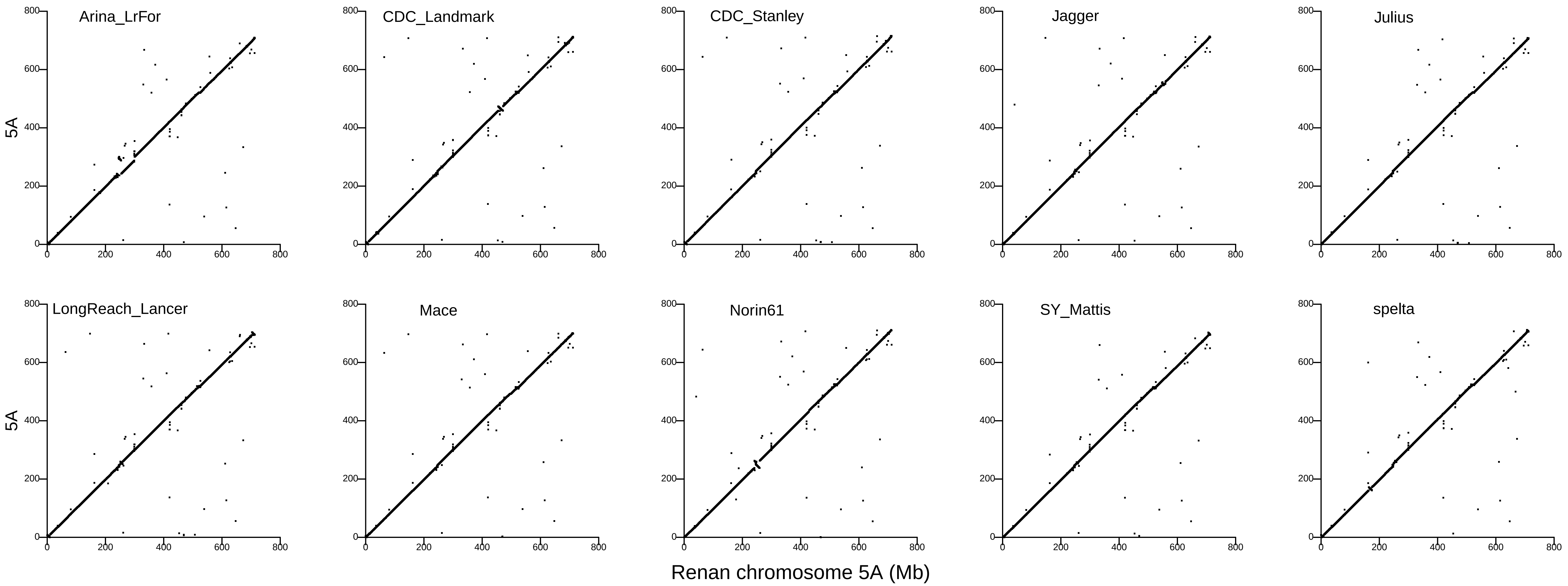
Arina_LrFor
CDC_Landmark
CDC_Stanley
Jagger
Julius
LongReach_Lancer
Mace
Norin61
SY_Mattis
spelta
5A
5A
0
200
400
600
800
Renan chromosome 5A (Mb)

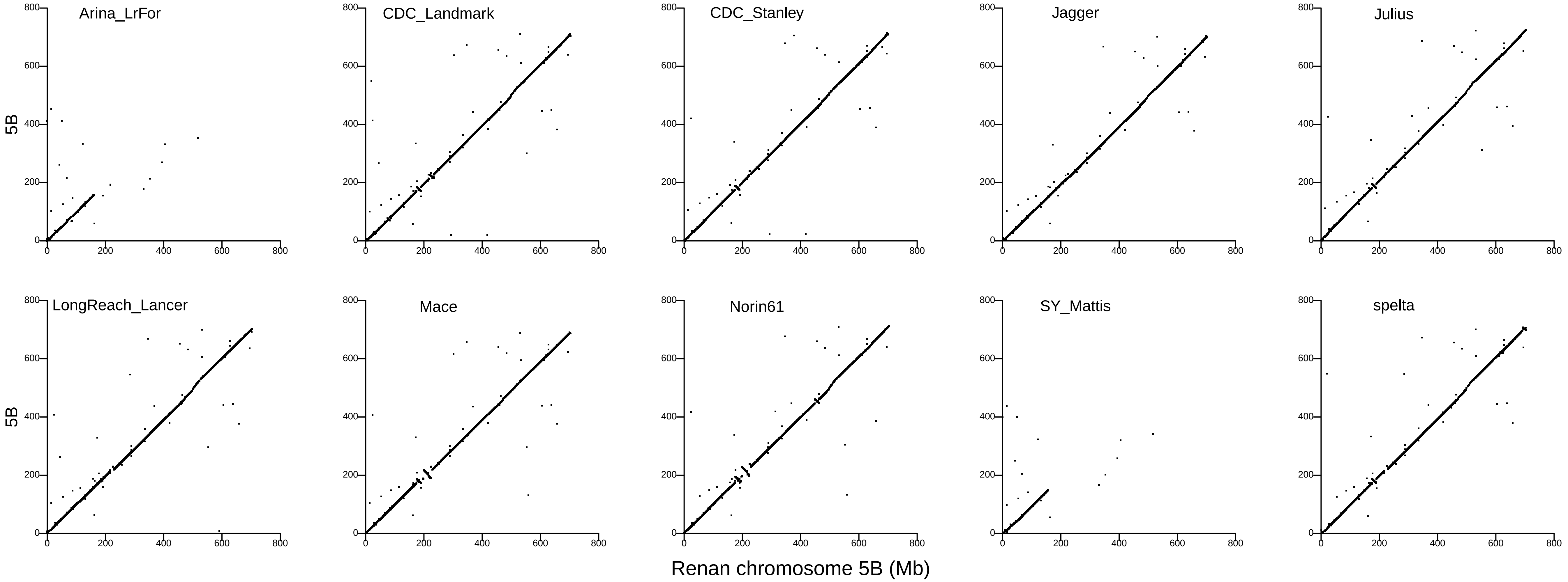
Arina_LrFor
CDC_Landmark
CDC_Stanley
Jagger
Julius
LongReach_Lancer
Mace
Norin61
SY_Mattis
spelta
5B
5B
0
200
400
600
800
Renan chromosome 5B (Mb)

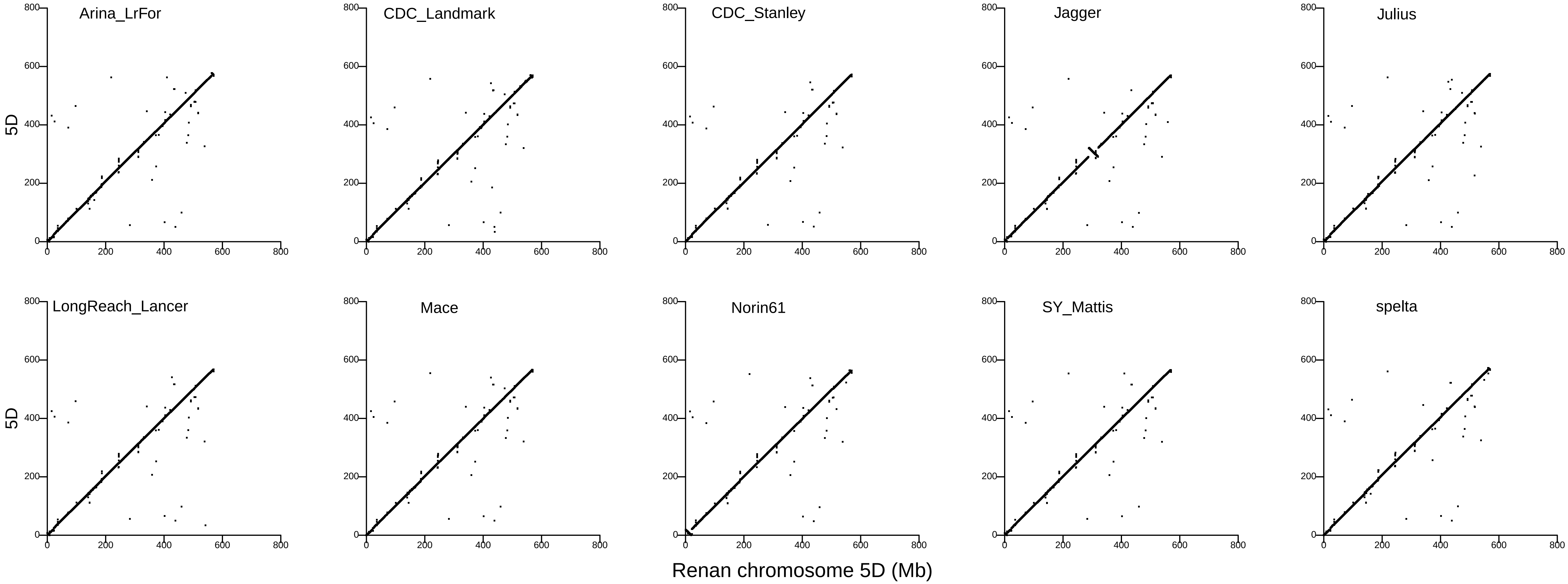

Arina_LrFor
CDC_Landmark
CDC_Stanley
Jagger
Julius
LongReach_Lancer
Mace
Norin61
SY_Mattis
spelta
5D
5D
0
200
400
600
800
Renan chromosome 5D (Mb)

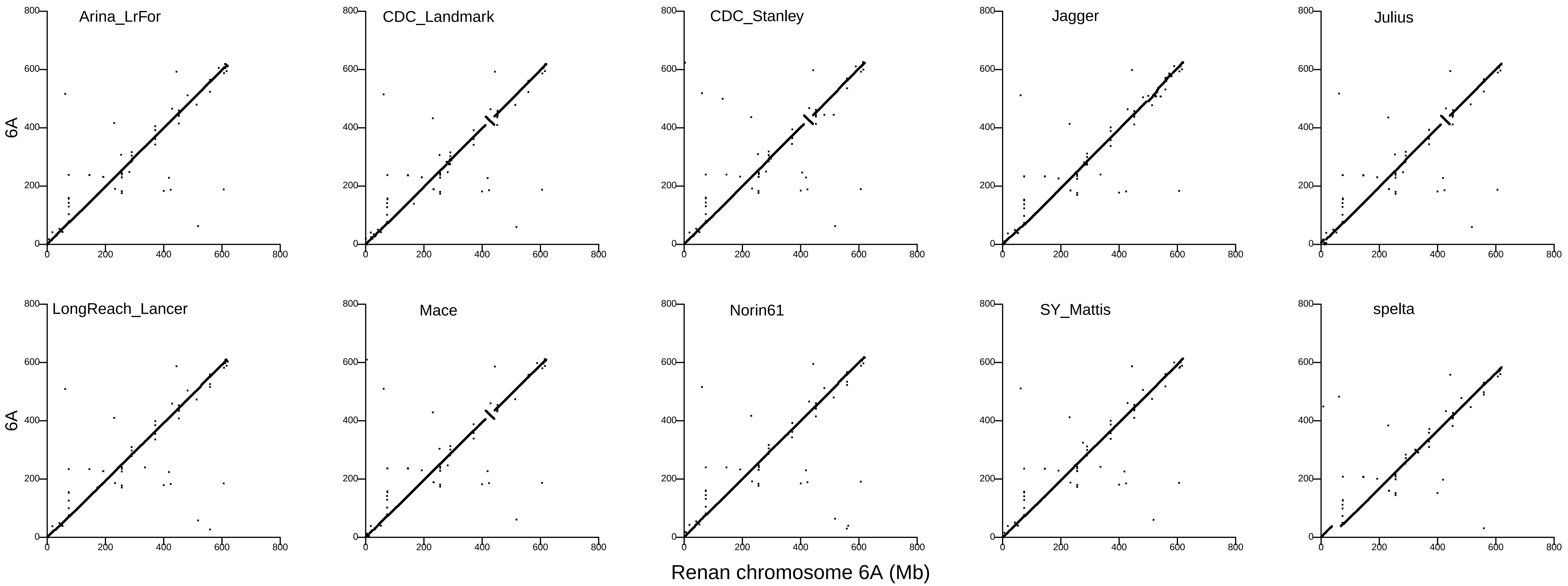
Arina_LrFor
CDC_Landmark
CDC_Stanley
Jagger
Julius
LongReach_Lancer
Mace
Norin61
SY_Mattis
spelta
6A
6A
0
200
400
600
800
Renan chromosome 6A (Mb)

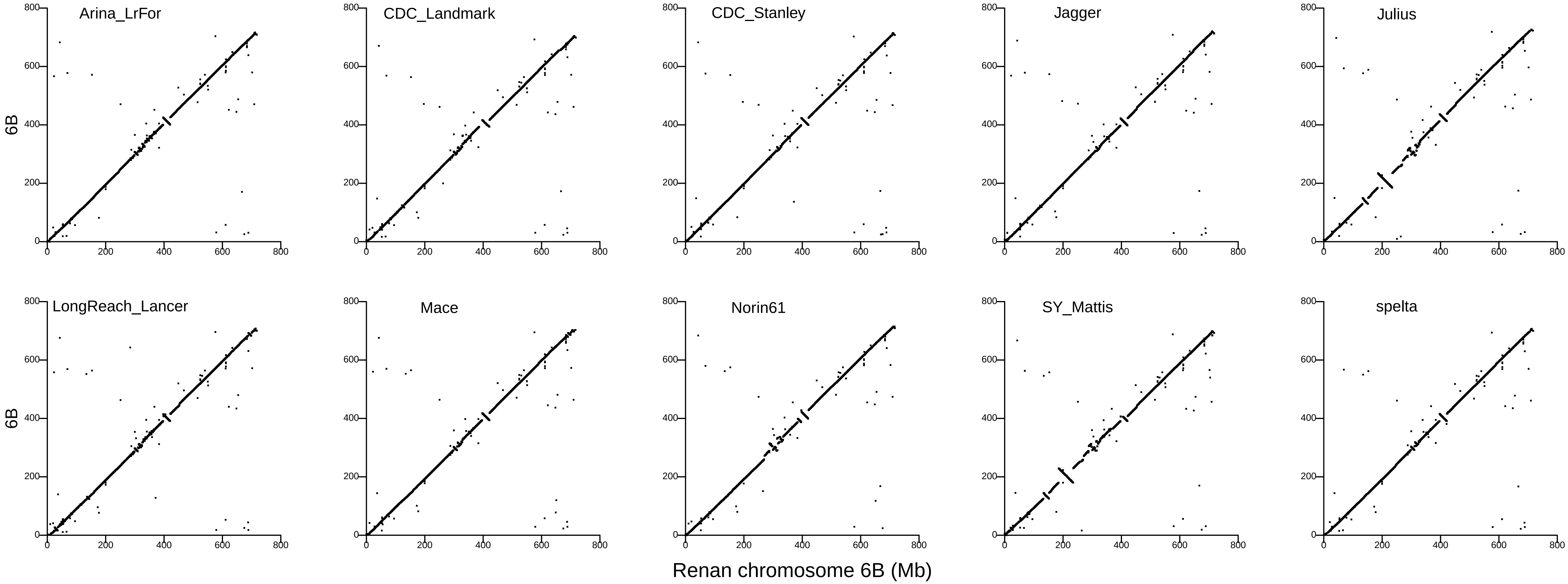

Arina_LrFor
CDC_Landmark
CDC_Stanley
Jagger
Julius
LongReach_Lancer
Mace
Norin61
SY_Mattis
spelta
6B
0
200
400
600
800
Renan chromosome 6B (Mb)

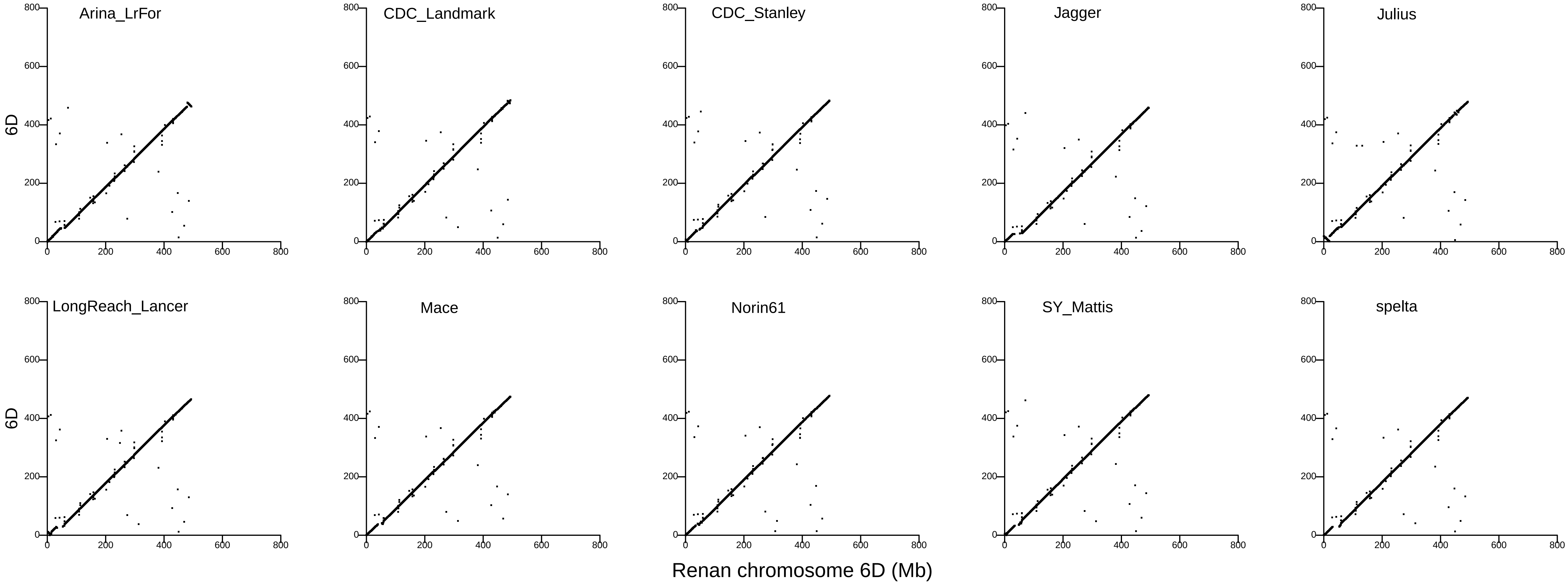

Arina_LrFor
CDC_Landmark
CDC_Stanley
Jagger
Julius
LongReach_Lancer
Mace
Norin61
SY_Mattis
spelta
6D
6D
Renan chromosome 6D (Mb)
0
200
400
600
800

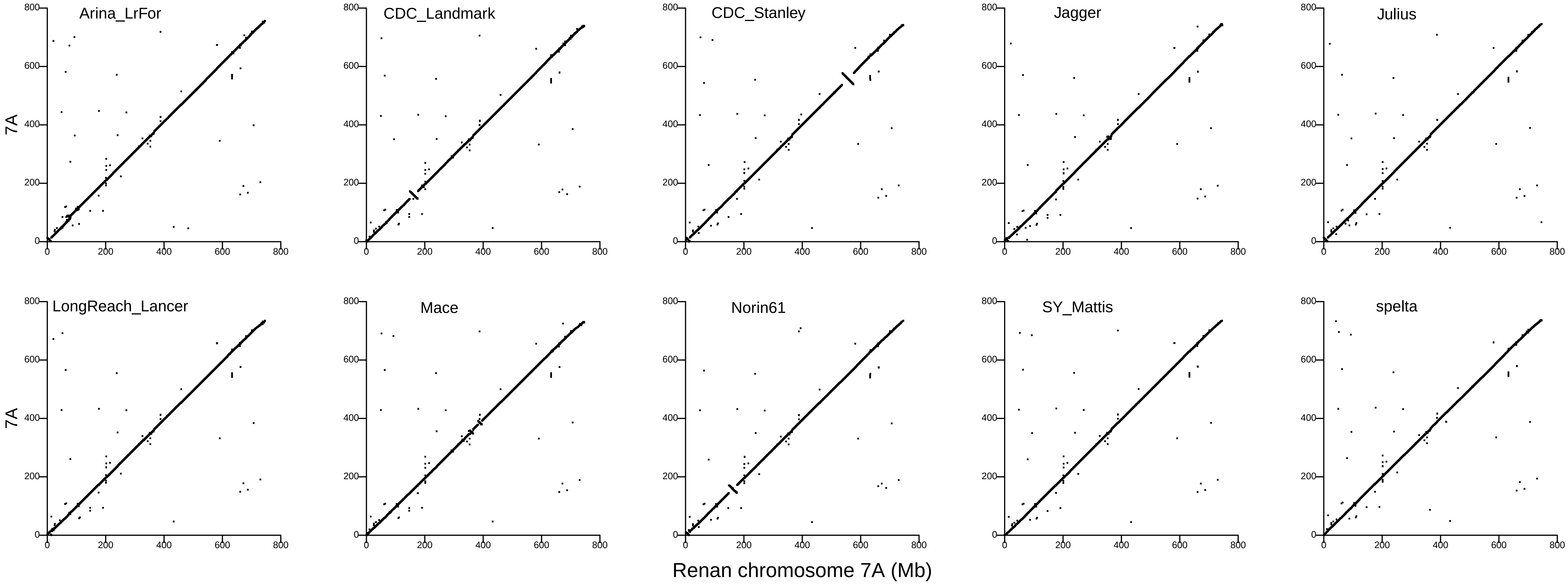

Arina_LrFor
CDC_Landmark
CDC_Stanley
Jagger
Julius
LongReach_Lancer
Mace
Norin61
SY_Mattis
spelta
7A
Renan chromosome 7A (Mb)

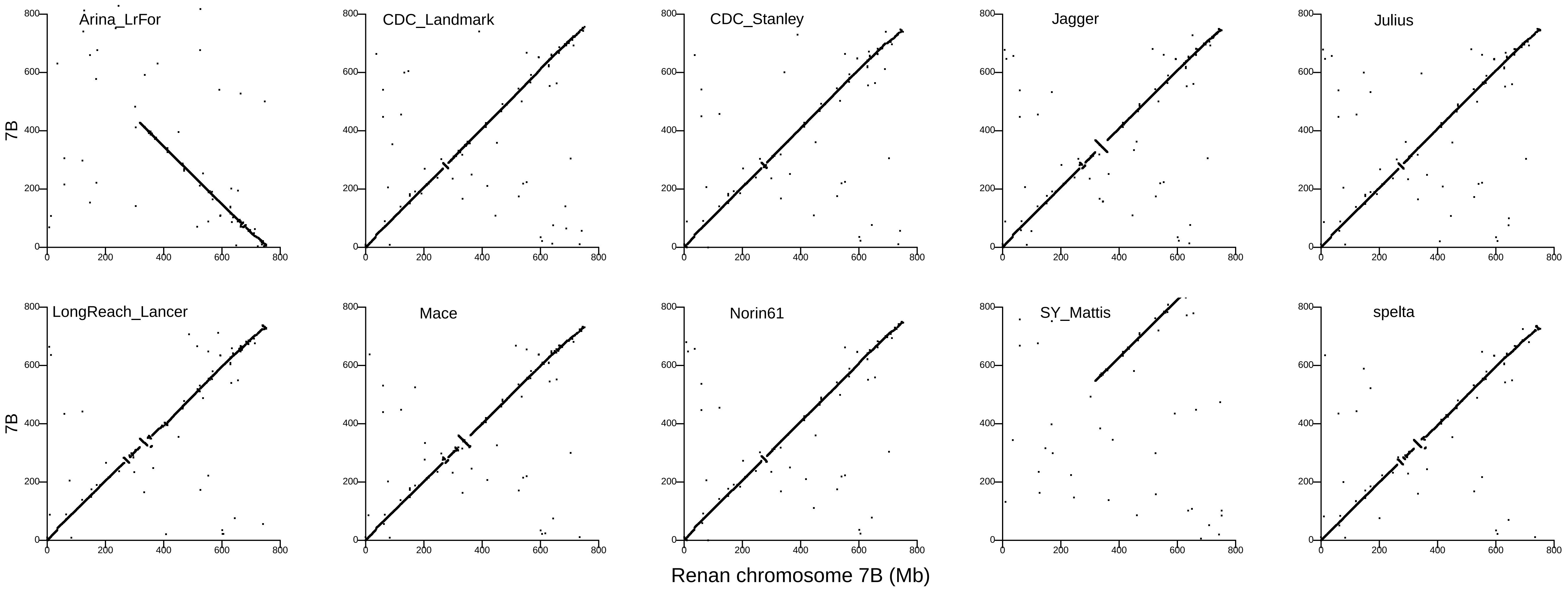
Arina_LrFor
CDC_Landmark
CDC_Stanley
Jagger
Julius
LongReach_Lancer
Mace
Norin61
SY_Mattis
spelta
7B
Renan chromosome 7B (Mb)
0
200
400
600
800

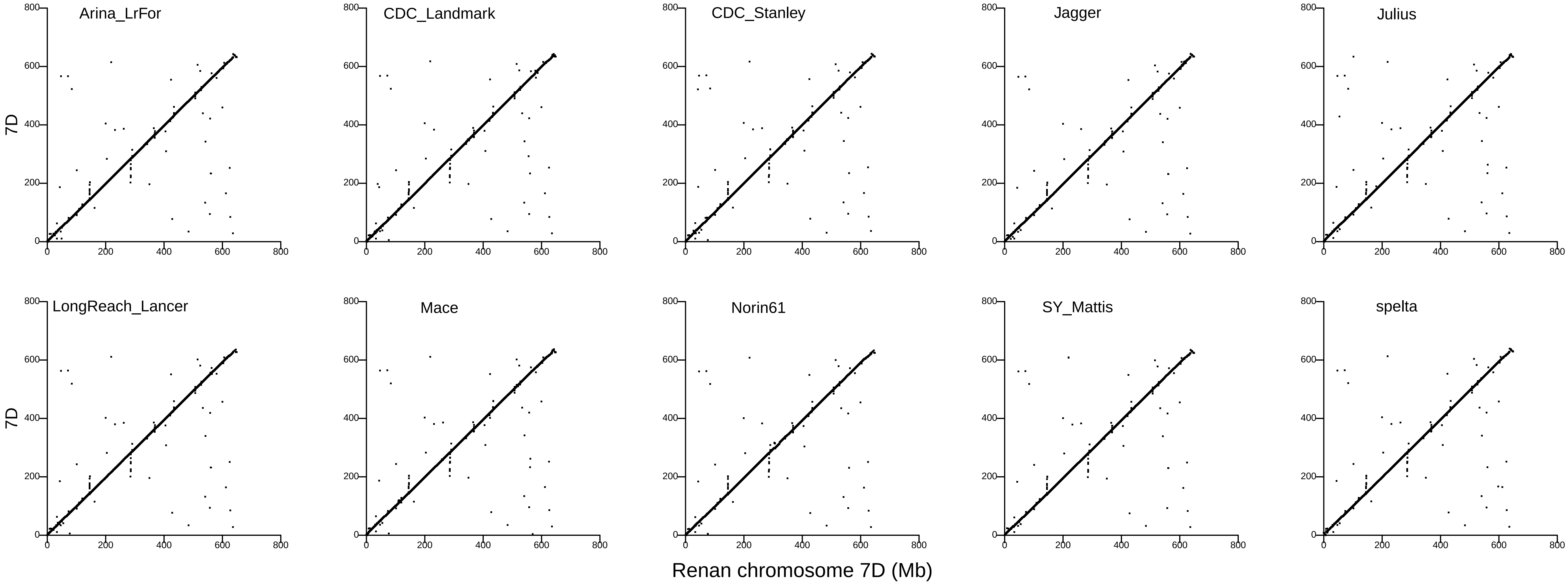
Arina_LrFor
CDC_Landmark
CDC_Stanley
Jagger
Julius
LongReach_Lancer
Mace
Norin61
SY_Mattis
spelta
7D
7D
Renan chromosome 7D (Mb)
