# Supplementary material for: Long-read and chromosome-scale assembly of the hexaploid wheat genome achieves high resolution for research and breeding
Source: Gigascience. 2022 Apr 28;11:giac034. doi: 10.1093/gigascience/giac034 (PMC9049114; doi:10.1093/gigascience/giac034)
Supplement: giac034_Response_to_Reviewer_Comments_Original_Submission [file giac034_response_to_reviewer_comments_original_submission.pdf]

Author's Response To Reviewer Comments

Close

==First of all, we would like to thank the three reviewers for their meticulous reading of our article. We have taken into account their comments, and believe that this new version of the manuscript is now of better quality. To make the review process easier, all the changes we made are in blue in the main article.==

Reviewer #1:
Comment 1:
The authors could more clearly and accurately present and discuss sequencing and assembly approaches, including the advantages and limitations of the ONT assembly presented here. While the standards of 'quality' for assemblies are evolving, there are standard sets of 'science-based' criteria for considering the quality of a genome, such as the 14 criteria listed in the manuscript here. Many of these criteria are ambitious, particularly for wheat due to its size and complexity, and many criteria are not met using previous assembly approaches, or the approaches used in this study.
==We thank the reviewer for this comment. We extended our evaluation of the Renan assembly with software used in the cited article, when applicable to the wheat genome. The quality of the consensus was evaluated using the Merqury software with Illumina short-reads. To perform the same analysis on Chinese spring, we downloaded short reads from the ENA repository (PRJNA393343). As expected, the contiguity is in favor of the Renan assembly whereas the base-accuracy is higher for the short-read assembly. We added the NG50, NG90 (at contig and chromosome levels) and the quality score in the section “Quality assessment of the assembly” and in Table 1. We think these new results now clearly present the advantages and limitations of the ONT assembly of Renan.==

It is true that CS and 10+ Wheat Genomes do not use long reads; however, these assemblies are valuable and have been rigorously validated using 10X Genomics, Hi-C, and long read data. They also perform well for TE content, BUSCO (as outlined by Tables 1 and 2 and Fig 3 in this manuscript), and they were actually used in this MS as a reference for guiding the ONT assembly. I would also expect that they have a better base pair accuracy than the assembly presented here. I therefore suggest that the authors revise their statement "these assemblies have been produced using short-read technologies and are therefore not up to the quality standard of current genome assemblies".
==We agree with the reviewer that the existing short-read assemblies are valuable, we just wanted to emphasize the contrast in terms of contiguity and number of gaps with assemblies based on long-read technologies. We have replaced the sentence which could be misinterpreted by “While being a valuable and highly validated resource using multiple technologies, these assemblies were produced using short-read technologies and therefore may contain a higher number of gaps compared to genomes assembled with long reads”.==

If the authors wish to discuss assembly quality, which I recommend they should, I suggest focusing on advantages and limitations of each technology and assembly approach in a constructive way, perhaps with a stronger focus on the value of the ONT resource developed here. In regards to base pair accuracy, ONT is at a disadvantage to short reads or to PacBio. This is particularly true in the context of HiFi reads, which have increased accuracy over ONT and Illumina and have greater lengths than Illumina, but PacBio and HiFi are not discussed. This is not to say that PacBio is superior in every way, the reads from ONT are longer and these hold a significant value. As an example of differences between PacBio and ONT that might provide useful context to describe the differences between ONT and PacBio approaches, please see:
https://pubmed.ncbi.nlm.nih.gov/33319909/, for differences between short read (TriTex) and PacBio, please see https://www.nature.com/articles/s41586-020-2947-8 . All of these approaches are valuable but have both advantages and limitations, with ONT also having many clear advantages and disadvantages. But these need to be clearly communicated and supported, either through the results of this study or through the literature. For example, in the discussion, the authors state that "ONT devices HAVE a real advantage over other long-read technologies". There is only one other long read sequencing technology, so are if you saying that ONT HAS a 'real advantage' over PacBio based on read length, this is valid, but can be stated more explicitly and with examples of the read lengths from this study and the

literature. It is then stated that the "error rate is drastically reduced for nanopore", again this valuable and a great advancement in regards to ONT, but it would be wise not to dismiss that this error rate is still higher than PacBio HiFi, which again can be stated explicitly with support from the literature.
==In fact we wanted to avoid a comparison of the two long-read technologies, ONT and PACBIO. But we agree that the first paragraph of the discussion was too vague and we decided to be more explicit. We have now compared the two technologies in regard to our results and other results from the literature. We hope that now this part is clearer and more explicit.==

While both of these concepts are important, after they are stated, they are not actually discussed or framed to highlight the work from this study. The true advantage of ONT, even over PacBio HiFi, is that the long reads can resolve more complex regions that span greater distances, which are abundant in wheat (see reference from above). The authors are presenting an exciting and valuable resource with this genome assembly and this assembly has advantages due to the application of ONT, for the reasons mentioned above regarding long complex regions, but these are not fully highlighted and the authors do not take full advantage of what this assembly has to offer. I think the authors should provide additional context and support related to the value and drawbacks of their ONT assembly. The advantages are discussed superficially at the gene level through a couple of examples (Fig 5), though none of these examples are supported with any significant biological data or validation analysis. There are many interesting features of genomes that are captured by ONT that are not captured well by short reads or PacBio, and it is unfortunate that these are not explored in any significant depth in the manuscript.
==The main objective of this article was initially to provide rapid access to this new resource to the community, then to perform the biological analysis of the Renan genome as a second step. Moreover, showing that an assembly is better than another in a given region is not trivial if the work has been conducted on two different cultivars. However, we agree that the validation and the presentation of the Renan assembly is an important feature of this article. We have added several analyses and we hope that the manuscript is now beyond a “data note” article. For that purpose, (i) we extended our evaluation of the Renan assembly using the metrics from the VGP project, as mentioned above; (ii) we validated the example of Figure 5 using the optical map and show a good concordance between the maps and the assembly in this region; (iii) we performed new analyzes to characterize haplotypic blocks so that users of our assembly can be informed of Renan’s positioning relative to other wheat lines, (iv) additionally these analyzes allow us to screen the genome for introgressions and validate these regions in the Renan assembly using the optical map, and (v) finally, we performed a comparison of the genomic region which contains the Sm1 gene (a gene in wheat known to provide resistance to the orange wheat blossom midge, OWBM) in each line and showed that Renan carry the same Sm1-haplotype than CDC Landmark which is resistant to the OWBM.==

Comment 2: Some of the 'highlighted features' in the manuscript could be better selected/executed. This comment relates to the previous comment on having little detail on what the ONT genome is uniquely capable of providing over other approaches. Instead, the authors focus on some anomalies in the D genome as well as differences in the nanopore software for base calling. It is unclear to me what the objective is of the report on the D genome. I suspect that this may be due to differences in repeat content between D and the other subgenomes, or an artifact of the tools and analyses used. Page 6, Figures S1 and S2, may be a consequence of poor read filtering for reads that align ambiguously - i,e perhaps reads from A and B may crossmap at a greater likelihood than those from D due to differences/similarities in repeat content between subgenomes. Once reads are aligned, the alignments should be properly filtered using standard 'best practices for NGS'- I do not see that any filtering or analysis of cross mapping was performed, but I may have missed it. Once the alignments are filtered, read coverage dips and peaks can then be assessed statistically using tools such as CNVnator and cn.mops, which are designed specifically for comparative read depth analysis since depth may not be normally distributed, rather than arbitrarily looking at 2 times the median. There may be differences between genes and intergenic regions in terms of mapping accuracy, so it may be ideal to interrogate read depth for those separately.
==Regarding the unexpected coverage, we took the advice of the reviewer and decided to delete this part from the manuscript. While we initially thought that warning users about cross-mapping issues using short-reads was reason enough to keep this part, we agree that filtering alignments can generally erase this artifact. We removed Figures S1 and S2 and the corresponding text in the article. We now present the coverage along the 21 chromosomes in Figure 1, and we used the mosdepth software with suitable parameters to remove the ambiguously mapped reads. We also kept the presentation of large CNVs, but this time they were predicted using the CNVnator software as recommended, and we only plot large events (>500Kb) in Figure 1 for clarity. We modified the text, the method section and the corresponding tracks in Figure 1, accordingly.==

The increased gaps is also interesting and I wonder if this could be due to the read accuracy of ONT and read mapping and assembly biases when having similar subgenomes. Nevertheless, the results and discussion on the D genome are interesting but distracting and likely reflect that the authors should take more time to explore their data and its biases before presenting this information. In summary, I believe that additional work is needed to bring value to the read depth and D genome analysis should the authors choose to include this in the manuscript.
==We thank the reviewer for this comment, and we were also surprised by the higher number of gaps in the D subgenome. This may be because the A and B subgenomes are closer together and maybe the D subgenome is more difficult to assemble even though it has a smaller genome size and contains less repetitive elements. We observed the same trend in another polyploid genome, the rapeseed and its two subgenomes A and C, where A is smaller and contains less repetitive elements but the assembly contiguity of the A subgenome is generally lower. We updated the text with this observation.==

While I agree that it would be useful to communicate that a significant gain was observed when basecalling using the more accurate basecaller, the emphasis on this is disproportionate to its value in the manuscript. The observation of a better assembly when using reads from a more advanced basecaller is not something new. As for the error rate of the ONT between organisms (yeast and wheat), with a sample size of 2, I do not think that this is worth presenting or discussing in any detail. While this may just be an artifact of the DNA quality itself from two experiments, I suspect that this may be a valid result from the manuscript and due to sequencing repeats, which are more abundant in wheat, in combination with how these basecallers self train to be more accurate. While this is certainly valid, it is not novel or interesting. This result comparing species was not tested with sufficient scientific rigor/evidence, it distracts from the central result of the manuscript, and just reaffirms something that we already known about the basecalling software and challenges of sequencing homopolymers and the importance of getting accurate reads using the more advanced basecalling methods.
==We agree with the reviewer that it is important to show that we observed a significant gain when using the more accurate basecaller. In addition, we believe it is important to show that the error rate of ONT reads may be higher than presented by the ONT company. We have used the yeast sequencing data to show that the error rate communicated by ONT is real but may not be representative of the general case. Homopolymers may be one case, but the methylation pattern is an important difference that affects the raw signal and it underlines the need for dedicated training sets in current basecallers. We have now organized this part of the manuscript differently.==

Comment 3: Why Renan?
This comment relates to the other two comments on the selected areas of focus. The biological story, which was on gliadins, was of some value and highlighted some of the advantages of an ONT assembly, but this was not supported by any significant biological data. Renan is a well-known cultivar with abundant genomic data, mapping populations, trait data for diseases, etc. It is unfortunate that the authors could not use the genome to dig deeper to more thoroughly demonstrate the value of this assembly specifically in the context of ONT and genomics of wheat or the biology of wheat and Renan, specifically. With abundant QTL data available specifically for Renan, these could have easily been anchored to the assembly to highlight novel transcripts from the RNAseq from this study, just as an example. Even the comparisons of the Renan assembly to other available assemblies was mostly superficial and did not highlight in significant detail the value of having an ONT assembly or the value of having data specifically for Renan. While a detailed 'biological story' may be beyond the scope of this manuscript, there was minimal effort to highlight the value of the assembly, and this comment is more of a larger reflection that more could have been done to highlight the value of the genome to support the author's vague claims that the genome "will benefit the wheat community and help breeding programs".
==We agree with this comment and add information of the interest of the Renan genotype at the end of the Introduction, as follow: "Here, we report the first hexaploid wheat genome based on ONT long-reads. We sequenced the genome of the French variety Renan, one of the most used varieties in organic farming. The Renan genome carries multiple resistance genes against fungal pathogens (leaf rust, stem rust, yellow rust, eyespot) originating from introgression of DNA regions coming from the wild species Aegilops ventricosa. [...]". ==

Minor Comments
The absence of numbered lines made it difficult to provide more detailed feedback, but there are minor items throughout, so I suggest numbering the lines and also giving the manuscript a thorough review.
==We have added line numbers to facilitate the review process.==

I appreciate that the authors present and suggest methods for future assembly of complex genomes

using ONT, but unlike the abstract states 'we also provide the methodological standards to generate high-quality assemblies of complex genomes'. I would argue that the standards used for ONT assembly are known and are not established here. I would also suggest caution when stating that the methods here should be considered the 'standard' for the reasons indicated in Comment 1 regarding other approaches used to assemble complex genomes, such as PacBio/HiFi, and the lack of a proper investigation/discussion/comparison of assembly quality.
==We agree with the reviewer and have changed the sentence in the abstract as follows: "We also provide a framework to generate high-quality assemblies of complex genomes using ONT".==

Page 2: last line - what is the abbreviation ca. ?
==ca. is a written abbreviation of circa (=about).==

Table 1: Busco is presented twice with different values.
==The two BUSCO results refer to the genome assembly and the gene prediction. The first was launched using the assembly as input and the "–mode genome" argument and the second using the fasta file of the predicted proteins and the "--mode proteins" argument. We updated Table 1 and believed the difference is now clearer.==

Table 1 and 2 use different versions of RefSeq, I would stick to one version.
==We thank the reviewer for pointing out this problem. We have now updated Table 1 which was previously based on an older version of the Chinese Spring assembly. ==

It is unclear to me what trend or result is that the authors are trying to present in figure 1, which I would say is common for circos plots. Presenting data 'for the sake of presenting it' is not terribly valuable and I would encourage the authors to use the figures to present a trend or result that is impactful. In addition, the data that is presented is not presented clearly, and is cryptic. The roman numerals in the figure caption for Figure 1 are not actually in the figure. The caption also indicate that the dots indicate lower and higher values, but not of what - perhaps density of gaps? The color scales are not presented for each track. Two of the color scale pallets also look similar.
==We agree with the reviewer that Figure 1 did not present an impactful result. However it seemed important to us to present the complex structure (three subgenomes and large size) of the wheat genome to non-specialists. We have now added the coverage with short and long reads, as well as the coverage with A. ventricosa reads. We have also added a way to easily identify each track and we have described the color scales in the legend.==

Page 6: 62% of exons were identical, which means 48% had SNPs, so the authors argue that SNPs are therefore rare at 48% of exons? I do not think that 48% of exons having SNPs is rare, I think it that this would mean that nearly half of exons have SNPs, so this is therefore common. Perhaps this statistic is misleading and the focus should instead be on the 0.7% divergence. How does this value compare with other within species comparisons of gene content and could this be an artifact of ONT accuracy? This question relates to a general comment that the authors could do better at bringing relevant comparisons or parallels in from the literature throughout the manuscript to bring value to any findings or insights they are presenting. Particularly in the context of other ONT assemblies.
==We agree that this conclusion (weak polymorphism) lacks some comparison with previous knowledge. The level of sequence conservation estimated at the whole genome level between wheat genomes was commented on in the comparative genomics study by Brinton et al. 2020 focusing on haplotype reconstruction. However, the information about the proportion of polymorphic genes was not mentioned in this paper.
In the revised version, we modified this paragraph to avoid concluding that SNPs are rare but rather comment on the fact that it confirms previous (Brinton et al.) conclusions on nucleotide divergence of wheat varieties.==

Page 7, capitalize the T for technology, it is part of the name of the company and is a proper noun. This is repeated elsewhere.
==Thanks for pointing it out, we've changed the text accordingly.==

Page 7: 'on wheat'? this statement could be written more clearly
==Thanks for pointing it out, we've changed the text accordingly.==

The way that the text is worded, it sounds like the basis for selecting the SmartDenovo assembly was the number of unknown bases, when I suspect it was actually a multitude of factors (BUSCO, gene or TE content, assembly stats, etc). While I do not question the selection of the assembly, I do suggest a

clearer presentation of the information.
==We have updated the manuscript and made it clear why we chose the SMARTdenovo assembly: "Based on these results (proportion of gaps and gene completion), the assembly produced by SMARTdenovo was selected".==

I appreciate that the authors presented the data from multiple assemblers, one of the concerns with ONT is that the read accuracy is low and this may lead to issues in assembly of complex polyploids with similar subgenomes. I suspect that based on this study, it is clear that this is a valid concern for some assemblers, but may have been overcome in others. Though none of this is explored or discussed. Again, is there any information in the literature contrasting assemblers that could provide insights into what you observed?
==Comparing assemblers is not an easy task, as the results are highly dependent on the selected organism. As an example, a recent study has been performed on a heterozygous and non-model organism (https://doi.org/10.1186/s12859-021-04118-3), but the conclusions they draw on the Flye and Redbean assemblers are different from what we got on the wheat genome. In our experience, the redbean assembler is not suitable for plant genomes.==

Searches at 90% identify and coverage for genes and TEs is not strict, especially with genomes that have highly identical subgenomes. If you reduce your thresholds enough, all features will map to your genome…..
For the quality assessment, we searched for the presence of a gene dataset (i.e. the whole HC genes of Chinese Spring) by using BLASTN of individual exons (>30 bps). This avoids methodological problems due to spliced alignments. We agree that the 90%id-90%coverage threshold is somehow low to consider that the query exon is mapped at the orthologous position. To take this remark into consideration, we updated the analysis while considering a more stringent threshold of 95%id-95%coverage and updated the percentages in the manuscript. We found 96.2% of exons matching (instead of 97.6%), and the average %id between CS and Renan exons remained the same: 99.3%.
==For TEs, this is different. We used ISBP markers (150 bps) which correspond to the junction between a TE and its insertion site. The 150-mer is, thus, unique and we do not face problems due to multiple mapping here (as it is the case with gene copies). If an ISBP is fully aligned (90% overlap in case there is a SNP/indel close to the end of the 150-mer which makes the BLAST stop) at >90% identity, we can conclude the orthologous ISBP is present in Renan. In addition, a slight increase of this threshold did not change the results here since we just want to estimate a % of markers conserved between CS and Renan and found 94%, although we are aware that the 6% remaining does not mean there are 6% missing data in the assembly, but rather this represents true polymorphisms (PAVs) between the two genotypes. ==

The choice of language is often objective or not representative of the results. For example, the 'extremely' similar TE content between Renan and CS. Why not say it is similar and actually report a value or a % difference. This would be more concise and informative than using vague and overzealous language.
==We changed this sentence to consider this comment. Actually, using ISBP markers, which are sequences derived from TEs extremities, it was possible to assess that 94% of Chinese Spring markers were conserved in Renan. The variability that affects TEs between these two wheat genotypes is limited to a few percent. Thus, the proportion of each TE superfamily (Table 2) is almost identical to what we already described for Chinese Spring, simply because we annotated here a genome sharing >94% of its content with the CS genome. This is what we would like to make clear when writing "extremely similar". We corrected the language here, as suggested. ==

Page 8, short reads (dash or no dash?)
==We have edited the text and are now consistent throughout the manuscript.==

The font sizes in Figure 2 are very small.
==We have enlarged the font size and moved this figure to Supplementary data, as recommended by reviewer 2. This also addresses the fact that the emphasis on this part was disproportionate.==

The RNAseq is not really presented at all, except in the Materials and Methods. I thought the genes were ab initio predicted until I saw RNAseq in the materials and methods. I suggest at least making a note of RNAseq into the results and/or discussion since this additional effort does bring added value to the annotations and the manuscript. The discussion says de novo annotations, but I suggest explicitly stating that RNAseq was performed.
==It was stated in the Methods but in fact missing in the Results section. We have now clearly

mentioned that we used RNA-Seq data: “For that purpose, we produced RNASeq data for Renan from 28 samples corresponding to 14 different organs/conditions in replicates: grains at four developmental stages (100, 250, 500, and 700 degree days) under heat stress and control conditions, stems at two developmental stages, leaves at three stages, and roots at one stage), representing on average 78.8 million read-pairs per sample i.e 2.2 billion read-pairs in total”. Additionally, we have inserted dedicated parts in the Methods section to describe RNA extraction and sequencing and Table S8 lists the 28 samples.==

Figure 3 C and D do not have horizontal axis labels, the top should be labelled as subgenome, bottom as chromosome, and the vertical axis (not the top) should be labelled as number of gaps and chromosome length. Same comment for labelling of vertical axis for panels A and B, horizontal axis should be labelled as genome assemblies, which are reflected in the pallet/legend. Note that many of the colours in this pallet are similar and difficult to differentiate, it may actually take less space to label the bars with each wheat line to make it less cryptic.
==We modified the labels and added labels for easier viewing of each wheat line, and have updated Figure 3.==

How were the dotplots in figure 4 generated? Perhaps I missed it in the materials and methods. Also one of the axis have labels or units, etc.
==Dotplots were generated via mapping of ISBP markers (150 bps) designed in Chinese Spring. We have more than 4 million ISBPs and ISBPs are uniquely mappable sequences. These two features (high density + uniquely mappable) are well suited to compare large genomes while avoiding problems due to either, multiple mapping of repeats, and presence of low gene density regions. We mapped ISBPs using BWA while allowing mismatches but keeping only fully mapped ISBPs as representing orthologous loci between two genotypes. For dotplots, we plotted the positions of orthologous ISBPs.
We wrote this in the section “Comparison of genome assemblies” of the Methods. Here is the corresponding sentence: “We built dotplots between Renan, CS and 10 other reference quality genomes [...] by using orthologous positions of conserved ISBPs (1 ISBP every 2.5 kb on average) identified by mapping them with BWA-MEM (maximum 2 mismatches, 100% coverage and minimal mapping quality of 30).==

Much of the text in Figure 5 is too small and illegible.
==We have enlarged the font size in Figure 5.==

Page 10: The discussion is superficial and vague and should provide an accurate and pragmatic discussion of the results in the context of the literature. For example, the manuscript boasts a 'higher resolution'… but of what? Perhaps 'complex repetitive regions'? To reiterate my previous comment on the lack of literature support throughout the manuscript - Were these 'higher resolutions' of comparable to what was observed in the literature when ONT was applied to other systems? Again, these advantages of ONT and the assembly could be more thoroughly explored.
==In the light of our new findings and reviewer feedback, we have changed the discussion and added more litterature support. We hope the changes will make the discussion more precise and impactful. ==

Reviewer #2:
The authors report on a new assembly of a French wheat variety, Renan, using Oxford Nanopore sequencing technology combined with short read polishing, Bionano optical maps and Hi-C to validate chromosome-level ordering after anchoring to IWGSC RefSeq v2.1. This is the first study I know of to use Oxford Nanopore to assemble a complete wheat genome, and the results demonstrate that this technology (together with short read polishing, Bionano, Hi-C, etc) can be successfully applied to such a complex genome. Evidence is presented to support the quality of the assembly, but it is mostly at the global statistics level (eg: contig N50, total size of gaps) or macro-scale (whole chromosome dotplots). One detailed comparison between Renan and Chinese Spring of a biologically important region is presented.

The assembly is clearly of a high standard and is a valuable addition to the growing set of wheat varieties assembled to chromosome-scale. However, given the high quality of the IWGSC RefSeq v2.1 assembly (Zhu et al. (2021)), the claim that this assembly "achieves higher resolution for research and breeding" is quite strong and needs to be supported by more evidence. Given what is presented here, a more accurate statement might be "achieves higher contiguity and local completeness".

==We agree with the reviewer that the term "higher" is a bit strong. We have changed to "high" to be more in tune with the already high-quality of the existing genome assemblies.==

The high contig N50 of 2.2Mb is highlighted but I feel that more work is needed to demonstrate that the sequence is free of artefacts. The authors show in Figure 2 that this assembly has the lowest (though only slightly) complete BUSCO score out of the wheat genomes they compare with. Is it possible that some regions cause problems for the Oxford Nanopore technology and are either fragmented or completely absent from the assembly? Bionano maps were used but no evidence is presented to show the level of agreement with the assembled sequence and Bionano maps, as is done in Zhu et al. (2021).

In summary I think there are two key things to address:
1) More evidence supporting that the assembly is locally accurate, especially validation with alignment to Bionano maps;
We thank the reviewer for this comment. We validated assembly completion using genes and TEs from the Chinese Spring reference genome and demonstrated the high level of completion of our assembly. The optical map was used to validate the contigs produced by SMARTdenovo. The Bionano software cuts the contigs that appear chimerical, and this only slightly impacted the N50 contig (1.07Mb compared to 1.16Mb). As recommended, we have now shown examples of the agreement of the assembly with the optical map in several regions (Figure 5, 7 and 8).

2) Some results presented to relate this assembly to the existing chromosome-scale assemblies of wheat genomes.

To address these points, I think the following would greatly enhance the paper:
a) Using any method (eg: the method in Brinton et al. (2020)), identify identical-by-state haplotypes between Renan and Chinese Spring and the chromosome-scale assemblies from Walkowiak et al. (2020). This analysis would essentially produce a table which would be valuable supplementary data. A figure similar to Figure 3 (b) from Walkowiak et al. (2020) for a single chromosome, showing the regions of the existing wheat genomes sharing haplotypes with Renan would help place this genome into context.

b) This then defines large regions of the Renan assembly that can be directly compared at the base level to other assemblies. Select 2 or 3 examples to show how the Renan sequence compares to the equivalent region in other assemblies, and show the Bionano validation of Renan sequence together with presence of genes and gaps in each assembly being compared. Since the sequences being compared here should be the same (based on the previous step above), the genes from the Renan annotation can be mapped across and directly compared. This would provide direct evidence for the higher quality assembly being claimed.
==The main objective of this article was initially to provide rapid access to this new resource to the community, then to perform the biological analysis of the Renan genome as a second step. In addition, showing that an assembly is better than another in a given region is not trivial if the work has been conducted on two different cultivars. However, we agree that the validation and the presentation of the Renan assembly is an important feature of this article. We have added several analyses and we hope that the manuscript is now beyond a "data note" article. For that purpose, (i) we extended our evaluation of the Renan assembly using the metrics from the VGP project, as mentioned above; (ii) we validated the example of Figure 5 using the optical map and show a good concordance between the maps and the assembly in this region; (iii) we performed new analyzes to characterize haplotypic blocks so that users of our assembly can be informed of Renan's positioning relative to other wheat lines, (iv) additionally these analyzes allow us to screen the genome for introgressions and validate these regions in the Renan assembly using the optical map, and (v) finally, we performed a comparison of the genomic region which contains the Sm1 gene (a gene in wheat known to provide resistance to the orange wheat blossom midge, OWBM) in each line and showed that Renan carry the same Sm1-haplotype than CDC Landmark which is resistant to the OWBM.==

Figure 5 is a good comparison of a biologically important region, but it is unclear if the region in Chinese Spring and Renan is the same haplotype or not. This needs to be clarified at the start of this section. If the same, then the comparison is of two regions expected to be basically identical (and could be one of the examples used in the proposed comparison analysis above); if different, then that needs to frame the discussion since the region in Chinese Spring could theoretically contain different genes or more repeats, for example.
==We thank the reviewer for this comment. Indeed this is the perfect example where it is difficult to compare haplotypes. The Chinese Spring assembly contains more gaps and it is not excluded that some

genes repeated in tandem are missing. In this Figure, we have highlighted the fact that the omega genes which contain a microsatellite, systematically contain a gap in the CS reference sequence in comparison to Renan (Figure 7b). We did not focus on the number of copies but on the presence of gaps in these particular genes. This is also represented by the fraction of proteins that we were able to align on each genome assembly (Figure 7c). As suggested by the reviewer, we have added validation of this region using the optical map. Additionally, we focus on another locus common to a cultivar that was assembled using short reads and Renan (Figure 8). This example clearly shows the difference between short- and long-reads assemblies.==

Centromeres are not mentioned, though it is known to be a particularly difficult region in wheat genome assemblies. How do the centromeres look in this assembly and how do they compare to previous wheat assemblies? Do the Bionano maps validate the assembly in the centromere region? The analysis in point a) above would identify centromeres in common with other assemblies.
==Centromeric regions of wheat are not more complex than other parts of the genome, and these regions were already well captured using short-reads technologies. Dot-plots with other cultivars show a great agreement between assemblies.==

Likewise, the distal ends of chromosome arms, including the telomere sequences, are known to cause problems for Hi-C ordering and orientation. Again, the Bionano alignments demonstrating correct ordering would be particularly valuable.
==We examined the presence of telomeric repeats in the 21 chromosomes, and found that chromosome 7A had telomeric repeats at both ends. We validated both ends of the chromosome sequence with the optical map and added an additional figure (Figure S1) describing this comparison. ==

Figure 2 should be a supplementary figure.
==We followed the recommendation and moved Figure 2 to Supplementary data.==

Reviewer #3:
Aury et al have assembled the French bread wheat cv. Renan using Oxford Nanopore long read technology, optical map and Hi-C. They achieved a decent N50 of 2.2 Mb and constructed pseudomolecules with reference-guided approach. The assembly was corrected with Hi-C map. They annotated ~ 84% of repeats and projected gene models from previously assembled Chinese spring reference genome. The assembly quality was validated with standard approach. The Renan assembly showed good collinearity with existing short-read wheat assemblies and pinpointed some large (1 > Mb) inversions. There is a potential to catalogue structural variants i.e. large INDELs. However, many false-positives are expected when long and short read assemblies are compared. Nevertheless, they compared a complex tandem repeat region. They used appropriate tools for assembly and downstream analysis. This is an improved additional genome resource for wheat community.
==We thank the reviewer for this positive comment. We have added several analyses that will help the reader to decipher the impact of long reads on genome assembly. Moreover, the detection of introgressions represents important datasets and will help breeders to investigate the origin and genomic composition of modern cultivars.==

Close
