# Supplementary material for: Long-read and chromosome-scale assembly of the hexaploid wheat genome achieves high resolution for research and breeding
Source: Gigascience. 2022 Apr 28;11:giac034. doi: 10.1093/gigascience/giac034 (PMC9049114; doi:10.1093/gigascience/giac034)
Supplement: giac034_Response_to_Reviewer_Comments_Revision_1 [file giac034_response_to_reviewer_comments_revision_1.pdf]

Author's Response To Reviewer Comments

Close

Reviewer #1:
The revised manuscript addresses the major concerns/comments. The assembly and its report are an exciting new resource for the wheat community.
We thank the reviewer for this positive comment.

I only have one very minor comment below:
When writing variety names in text and figures, it is important to be exact because there are many varieties with similar names internationally. CDC Stanley, not "Stanley"; CDC Landmark, not "Landmark"; "LongReach Lancer", not "Lancer", not "LongRead Lancer" - typo on line 308. I suggest performing a thorough check throughout.
We have changed the variety names where necessary in the article and figures.

Reviewer #2:
Firstly, the revisions to the manuscript are a great improvement and help contextualise the assembly with other wheat genomes already published. The haplotype analysis allows identification of regions shared with other published wheat genomes to inform the comparisons presented.
The change in the title to "high resolution" is appropriate and more accurately reflects the results. I do wonder if Oxford Nanopore should be mentioned in the title since one of the main contributions of this article is to show the technology is capable of assembling the wheat genome to a high standard.
We agree but unfortunately the title is already too long, and the term long-reads, while not specific, is a good way to describe the Oxford Nanopore technology.

The main remaining concern I have is around the claim that this assembly provides the "most contiguous and complete" (stated in the abstract) assembly of bread wheat to date.
Objectively our assembly is the most contiguous to date, but we agree with the reviewer that the completeness is difficult to estimate accurately because different genotypes contain intrinsic differences. To do this reliably, one would need to sequence a given genotype twice using short or long reads, and that's not what this article is about, so we decided to remove the word "complete".

As Reviewer 1 also noted, what is needed is a consideration of the pros and cons of this ONT-based assembly compared to other approaches that have been published before. It is clear that this method is capable of producing a high quality assembly, but given the already high quality of wheat assemblies, what exactly does this approach do better and what are its limitations? Is higher contiguity with lower base-level accuracy preferable to high base-level accuracy with lower local contiguity (small gaps)?
This is a good question and the answer will depend on why users need a wheat genome assembly, what type of application they are using the genome sequence for. However, we have shown in the article, with the omega genes for instance, that now these particular genes do not contain any gaps in their coding sequence.

In this context the following points aim to highlight some remaining issues with suggestions on how to address them - in each case the aim is to be able to make a definite statement that informs the reader about the relative merits of this assembly.
We think that evaluation of a given assembly, while necessary, is an endless project. And above all, it remains difficult to highlight the merits of an assembly based on a comparison with different cultivars.

- The authors have added a useful "Base accuracy" metric to Table 1, which shows this assembly to score lower than IWGSC RefSeq v2.1 (which is mostly short-read based, with some areas replaced with Pacbio sequence). At the same time, if the lower quality is mainly due to problems in repeat regions, this may be acceptable to some users of the sequence, provided the gene space is highly accurate. The authors note (line 168) that "coding regions… may have higher precision." I feel this needs to be explored - one way to do this might be to also include the base accuracy analysis only on gene space (say including 5kb around the gene). This would enable a concrete statement to be made about the overall gene space accuracy.

To validate this hypothesis, we calculated the sequence quality of exons longer than 150 bases and obtained a QV of 35.9 (i.e. one error every 3.9Kb against one error every 2.0Kb in the whole genome). We have added this new result to the main text.

- The Bionano alignment shown in Figure 7d looks to me to show some ambiguity, with evidence of a collapsed and/or missing region, precisely within the dense cluster of genes. Without extra information here, given the Bionano alignment, it is hard to be certain that some genes have not been missed or that some genes could be collapsed/chimeric. Could RNA-seq alignments to the assembly be used to validate the gene models here? Or, given the size of the ONT reads, a small sub-region including a few genes could be shown together with the long and short reads aligning across it (presumably a single long read could include full length genes - which would be good evidence to back up the accuracy claims and show the value of long reads). This would enable a concrete statement to be made about the accuracy of these gene models in Renan.
This figure was used to investigate the coding sequence of the omega genes, which contain repetitive regions and are tandemly duplicated. The main result here is that many of these genes contain gaps in the Chinese Spring assembly, but not in the Renan one. We added a Supplementary Figure (Figure S5) that shows that long reads validate this region.

- Figure 8b aims to show the higher contiguity of the Renan assembly vs the equivalent region in Landmark. We can clearly see Renan has fewer gaps, but it's also clear that large regions, which are present in Landmark, are completely absent from the Renan assembly. The only comment about this is on lines 359-360 but given the claims of being "more complete", I feel it needs to be addressed more thoroughly. For example, mapping the genes in this region from Landmark to Renan, and the genes from Renan to Landmark (it has to be done both ways to avoid biases in the annotation method), and detailing any present in one and absent from the other, would at least give some biological context to consider, and enable a concrete statement to be made about coverage of the gene space in the two assemblies in this region. As it is presented, I find this example undermines the claim of completeness.
This is another example that underlines why it is complicated to compare genome assemblies of different cultivars. In the case of Renan, the gaps were due to short contigs that were difficult to organize using optical maps (due to an insufficient number of labeling). The size of gaps was estimated using the optical map, which usually provides an accurate estimate. By comparison, many gaps in the CDC Landmark region seem to be of unknown size, and have a 10bp length. We agree that figure 8b is misleading, and have decided to zoom in and show only the fragmentation around the Sm1 gene.

A few further points:
- Figure 7 needs the haplotype context around this region as shown in Figures 4, 5, 6 and 8. I am guessing the haplotype in this region is different between Renan and Chinese Spring, in which case the text around this figure should be clearer. As I read it I'm not sure if an identical region is being compared (in which case the two assemblies can be directly compared and conclusions made about their pros/cons) or different (in which case interpretation of the two assemblies is not as simple). If different, then it should be made clear that eg: 7b highlights the structural differences between haplotypes (to avoid potentially interpreting it as assembly inconsistencies). Also, the value of 7c is unclear since it is not surprising genes from Renan map well to itself and poorly to genes we already know have gaps. The legend for 7c should also make clear that it is the Renan genes being mapped to both regions (or if I've misunderstood, please clarify).
Haplotype blocks and visual karyotypes, including chromosome 1B, are all available on the github mentioned in the “Availability of supporting data” section. We have edited the text and explicitly indicated the availability of blocks on all chromosomes.
Indeed, the region that contains the storage protein gene cluster may be too divergent between cultivars (or may contain too many gaps) to be clustered. Orthologous regions in CS and Renan were identified using both ISBP markers and homeologs triad genes. But this analysis did not compare Renan and Chinese Spring with the expectation that they share the same haplotype. Here we study the structure of the coding genes and in particular the gaps in the genomic sequence that affect these important genes. Figure 7a shows an overview of the region with orthologous relationships between CS and Renan and Figure 7b is a zoom on the omega genes that contain a high number of gaps in CS. Maybe it was unclear, but in Figure 7c we used gene sequences annotated in the CS genome by Huo et al. (Front. Plant Sci., May 2018). And, as in Figure 7b, we show that gaps affect the coding sequences and disrupt alignments of omega gliadin-encoding genes between CS and Renan. We have clarified this point in the main text and updated the legend of Figure 7.

- Figure 8 describes the Sm1 locus and notes that Landmark and Renan are known carriers of the gene. But the haplotypes shown in 8a, in the roughly 1Mb around the gene, look quite different in the figure.

The text says: "This analysis revealed a strong similarity of the Sm1 locus between CDC Landmark and Renan (Figure 8a), the presence of the Sm1 gene in blocks shared between the two cultivars." ("including" should be added after the comma) (lines 355-256) How to interpret this? Is the Sm1 gene and surrounding sequence exactly the same in Landmark and Renan? If so, this should be shown/clarified and the apparent difference in haplotypes shown in 8a explained.
The surrounding blocks of the two regions have the same colors indicating good conservation only between Renan and CDC Landmark, but there are still some sequence variations that could explain the white blocks. Some chromosomes are highly conserved (like 6A or 2D) and others have greater variability (like 2B which carries the Sm1 gene). This is why comparing cultivars can be misleading.

- Figure 6a is a little confusing as it shows Ae. ventricosa reads covering most of 2D, but is presumably background noise, while the introgression has zero coverage. Could this be explained more clearly? Similarly for part b, the introgression has similar coverage to other parts of the chromosome which are not from Ae. ventricosa. Do the alignments need to be filtered more strictly?
Thank you for your comment. In fact, the Dv genome of Ae. ventricosa is evolutionarily close to the D genome of hexaploid wheat. As a result, non-zero coverage is expected and is not background noise. Moreover, the introgression region has zero coverage because this region is different from Ae. ventricosa Dv genome. We added this information in the legend of figure 6.

- Figure 6b, while interesting, is a distraction as it does not relate to Renan. I would argue to only include parts a and c, since they are good examples of a candidate introgression shared between Renan and other varieties (part a) and something unique to Renan (part c).
This is also the case in figure 5a, but we decided to report introgressions in all cultivars to show that our method could be used to easily spot introgressions.

- The section starting on line 325: All references to Figure 5 should be Figure 7.
Thanks for pointing it out, we changed the text accordingly.

- The labels on Figure 5d don't make sense (eg: 32.5M appears twice).
Thanks for pointing it out, we changed the figure accordingly.

- Figure S1 needs scales added.
Sequence coordinates have been added to Figure S1.

- Lines 322-324 and Figure S4 are not new information (reported in Walkowiak et al., 2020) and do not relate to Renan so I think they are a distraction.
Again, we used these known events to validate our method based on a colored de Bruijn graph and we prefer to keep the original text without modifications.

Close
