# Supplementary material for: Long-read and chromosome-scale assembly of the hexaploid wheat genome achieves high resolution for research and breeding
Source: Gigascience. 2022 Apr 28;11:giac034. doi: 10.1093/gigascience/giac034 (PMC9049114; doi:10.1093/gigascience/giac034)
Supplement: giac034_Response_to_Reviewer_Comments_Revision_2 [file giac034_response_to_reviewer_comments_revision_2.pdf]

Author's Response To Reviewer Comments

Close

Reviewer #2: Thanks to the authors for these revisions. I am satisfied with the responses to the points raised and now recommend this manuscript for publication. This resource is a great addition to the growing number of chromosome-scale wheat genome assemblies.

A couple of minor points:
- With regards the analysis presented in Figure 7: I understand now that the complete Chinese Spring protein sequences coming from Huo et al. (2018) were annotated against the Pacbio-based Zimin et al. (2017) assembly. This explains why they are more complete compared to the IWGSC assembly being compared here. To make it absolutely clear, I suggest wording lines 644-646 something like this: "Protein sequences of prolamin and resistance genes[35] annotated against the Pacbio-based assembly of Chinese Spring[5], were downloaded and aligned to the IWGSC RefSeq v2.1 and Renan assemblies using BLAT[48] with default parameters."
==> We have modified the text accordingly. The new sentence is now "Protein sequences of prolamin and resistance genes[35], annotated from a 1B chromosome locus in the Pacbio-based assembly of Chinese Spring[5], were downloaded and aligned to the IWGSC RefSeq v2.1 and Renan assemblies using BLAT[48] with default parameters."

- The authors only mention "IWGSC RefSeq v2.1" once in the text (line 589) - it is usually referred to as "CS RefSeq v2.1" (sometimes "Chinese Spring RefSeq v2.1") in the text or "CS" in legends and figures. I wonder if it should be explicitly referred to as IWGSC RefSeq v2.1 throughout or at least made clear somewhere that CS = IWGSC RefSeq v2.1. Either way it should be consistent.
==> We explicity mentionned at the beginning of the article that CS RefSeq v2.1 and CS refer to IWGSC RefSeq v2.1. The added sentence is "The first reference-quality genome sequence with a comprehensive annotation was published by the IWGSC in August 2018[7] for the accession Chinese Spring (CS), named in the text CS RefSeq v2.1 or simply CS."

Close
