# Supplementary material for: Long-read and chromosome-scale assembly of the hexaploid wheat genome achieves high resolution for research and breeding
Source: Gigascience. 2022 Apr 28;11:giac034. doi: 10.1093/gigascience/giac034 (PMC9049114; doi:10.1093/gigascience/giac034)
Supplement: giac034_Reviewer_1_Report_Original_Submission — Sean Walkowiak -- 10/15/2021 Reviewed [file giac034_reviewer_1_report_original_submission.pdf]

Reviewer Report

Title: Long-read and chromosome-scale assembly of the hexaploid wheat genome achieves high resolution for research and breeding

Version: Original Submission **Date:** 10/15/2021

Reviewer name: Sean Walkowiak

Reviewer Comments to Author:

Comment 1: The authors could more clearly and accurately present and discuss sequencing and assembly approaches, including the advantages and limitations of the ONT assembly presented here While the standards of 'quality' for assemblies are evolving, there are standard sets of 'science-based' criteria for considering the quality of a genome, such as the 14 criteria listed in the manuscript here: https://www.nature.com/articles/s41586-021-03451-0#Tab1. Many of these criteria are ambitious, particularly for wheat due to its size and complexity, and many criteria are not met using previous assembly approaches, or the approaches used in this study. It is true that CS and 10+ Wheat Genomes do not use long reads; however, these assemblies are valuable and have been rigorously validated using 10X Genomics, Hi-C, and long read data. They also perform well for TE content, BUSCO (as outlined by Tables 1 and 2 and Fig 3 in this manuscript), and they were actually used in this MS as a reference for guiding the ONT assembly. I would also expect that they have a better base pair accuracy than the assembly presented here. I therefore suggest that the authors revise their statement "these assemblies have been produced using short-read technologies and are therefore not up to the quality standard of current genome assemblies". If the authors wish to discuss assembly quality, which I recommend they should, I suggest focusing on advantages and limitations of each technology and assembly approach in a constructive way, perhaps with a stronger focus on the value of the ONT resource developed here. In regards to base pair accuracy, ONT is at a disadvantage to short reads or to PacBio. This is particularly true in the context of HiFi reads, which have increased accuracy over ONT and Illumina and have greater lengths than Illumina, but PacBio and HiFi are not discussed. This is not to say that PacBio is superior in every way, the reads from ONT are longer and these hold a significant value. As an example of differences between PacBio and ONT that might provide useful context to describe the differences between ONT and PacBio approaches, please see: https://pubmed.ncbi.nlm.nih.gov/33319909/, for differences between short read (TriTex) and PacBio, please see https://www.nature.com/articles/s41586-020-2947-8 . All of these approaches are valuable but have both advantages and limitations, with ONT also having many clear advantages and disadvantages. But these need to be clearly communicated and supported, either through the results of this study or through the literature. For example, in the discussion, the authors state that "ONT devices HAVE a real advantage over other long-read technologies". There is only one other long read sequencing technology, so are if you saying that ONT HAS a 'real advantage' over PacBio based on read length, this is valid, but can be stated more explicitly and with examples of the read lengths from this study and the literature. It is then stated that the "error rate is drastically reduced for nanopore", again this valuable and a great

advancement in regards to ONT, but it would be wise not to dismiss that this error rate is still higher than PacBio HiFi, which again can be stated explicitly with support from the literature. While both of these concepts are important, after they are stated, they are not actually discussed or framed to highlight the work from this study. The true advantage of ONT, even over PacBio HiFi, is that the long reads can resolve more complex regions that span greater distances, which are abundant in wheat (see reference from above). The authors are presenting an exciting and valuable resource with this genome assembly and this assembly has advantages due to the application of ONT, for the reasons mentioned above regarding long complex regions, but these are not fully highlighted and the authors do not take full advantage of what this assembly has to offer. I think the authors should provide additional context and support related to the value and drawbacks of their ONT assembly. The advantages are discussed superficially at the gene level through a couple of examples (Fig 5), though none of these examples are supported with any significant biological data or validation analysis. There are many interesting features of genomes that are captured by ONT that are not captured well by short reads or PacBio, and it is unfortunate that these are not explored in any significant depth in the manuscript.
Comment 2: Some of the 'highlighted features' in the manuscript could be better selected/executed
This comment relates to the previous comment on having little detail on what the ONT genome is uniquely capable of providing over other approaches. Instead, the authors focus on some anomalies in the D genome as well as differences in the nanopore software for base calling. It is unclear to me what the objective is of the report on the D genome. I suspect that this may be due to differences in repeat content between D and the other subgenomes, or an artifact of the tools and analyses used. Page 6, Figures S1 and S2, may be a consequence of poor read filtering for reads that align ambiguously - i,e perhaps reads from A and B may crossmap at a greater likelihood than those from D due to differences/similarities in repeat content between subgenomes. Once reads are aligned, the alignments should be properly filtered using standard 'best practices for NGS'- I do not see that any filtering or analysis of cross mapping was performed, but I may have missed it. Once the alignments are filtered, read coverage dips and peaks can then be assessed statistically using tools such as CNVnator and cn.mops, which are designed specifically for comparative read depth analysis since depth may not be normally distributed, rather than arbitrarily looking at 2 times the median. There may be differences between genes and intergenic regions in terms of mapping accuracy, so it may be ideal to interrogate read depth for those separately. The increased gaps is also interesting and I wonder if this could be due to the read accuracy of ONT and read mapping and assembly biases when having similar subgenomes. Nevertheless, the results and discussion on the D genome are interesting but distracting and likely reflect that the authors should take more time to explore their data and its biases before presenting this information. In summary, I believe that additional work is needed to bring value to the read depth and D genome analysis should the authors choose to include this in the manuscript. While I agree that it would be useful to communicate that a significant gain was observed when basecalling using the more accurate basecaller, the emphasis on this is disproportionate to its value in the manuscript. The observation of a better assembly when using reads from a more advanced basecaller is not something new. As for the error rate of the ONT between organisms (yeast and wheat), with a sample size of 2, I do not think that this is worth presenting or discussing in any detail. While this may just be an artifact of the DNA quality itself from two experiments, I suspect that this may be a valid result from the manuscript and due to sequencing repeats, which are more abundant in wheat, in combination with how these

basecallers self train to be more accurate. While this is certainly valid, it is not novel or interesting. This result comparing species was not tested with sufficient scientific rigor/evidence, it distracts from the central result of the manuscript, and just reaffirms something that we already known about the basecalling software and challenges of sequencing homopolymers and the importance of getting accurate reads using the more advanced basecalling methods.

Comment 3: Why Renan?

This comment relates to the other two comments on the selected areas of focus. The biological story, which was on gliadins, was of some value and highlighted some of the advantages of an ONT assembly, but this was not supported by any significant biological data. Renan is a well-known cultivar with abundant genomic data, mapping populations, trait data for diseases, etc. It is unfortunate that the authors could not use the genome to dig deeper to more thoroughly demonstrate the value of this assembly specifically in the context of ONT and genomics of wheat or the biology of wheat and Renan, specifically. With abundant QTL data available specifically for Renan, these could have easily been anchored to the assembly to highlight novel transcripts from the RNAseq from this study, just as an example. Even the comparisons of the Renan assembly to other available assemblies was mostly superficial and did not highlight in significant detail the value of having an ONT assembly or the value of having data specifically for Renan. While a detailed 'biological story' may be beyond the scope of this manuscript, there was minimal effort to highlight the value of the assembly, and this comment is more of a larger reflection that more could have been done to highlight the value of the genome to support the author's vague claims that the genome "will benefit the wheat community and help breeding programs".

Minor Comments

The absence of numbered lines made it difficult to provide more detailed feedback, but there are minor items throughout, so I suggest numbering the lines and also giving the manuscript a thorough review.

I appreciate that the authors present and suggest methods for future assembly of complex genomes using ONT, but unlike the abstract states 'we also provide the methodological standards to generate high-quality assemblies of complex genomes'. I would argue that the standards used for ONT assembly are known and are not established here. I would also suggest caution when stating that the methods here should be considered the 'standard' for the reasons indicated in Comment 1 regarding other approaches used to assemble complex genomes, such as PacBio/HiFi, and the lack of a proper investigation/discussion/comparison of assembly quality.

Page 2: last line - what is the abbreviation ca. ?

Table 1: Busco is presented twice with different values.

Table 1 and 2 use different versions of RefSeq, I would stick to one version.

It is unclear to me what trend or result is that the authors are trying to present in figure 1, which I would say is common for circos plots. Presenting data 'for the sake of presenting it' is not terribly valuable and I would encourage the authors to use the figures to present a trend or result that is impactful. In addition, the data that is presented is not presented clearly, and is cryptic. The roman numerals in the figure caption for Figure 1 are not actually in the figure. The caption also indicate that the dots indicate lower and higher values, but not of what - perhaps density of gaps? The color scales are not presented for each track. Two of the color scale pallets also look similar.

Page 6: 62% of exons were identical, which means 48% had SNPs, so the authors argue that SNPs are

therefore rare at 48% of exons? I do not think that 48% of exons having SNPs is rare, I think it that this would mean that nearly half of exons have SNPs, so this is therefore common. Perhaps this statistic is misleading and the focus should instead be on the 0.7% divergence. How does this value compare with other within species comparisons of gene content and could this be an artifact of ONT accuracy? This question relates to a general comment that the authors could do better at bringing relevant comparisons or parallels in from the literature throughout the manuscript to bring value to any findings or insights they are presenting. Particularly in the context of other ONT assemblies.
Page 7, capitalize the T for technology, it is part of the name of the company and is a proper noun. This is repeated elsewhere.
Page 7: 'on wheat'? this statement could be written more clearly
The way that the text is worded, it sounds like the basis for selecting the SmartDenovo assembly was the number of unknown bases, when I suspect it was actually a multitude of factors (BUSCO, gene or TE content, assembly stats, etc). While I do not question the selection of the assembly, I do suggest a clearer presentation of the information.
I appreciate that the authors presented the data from multiple assemblers, one of the concerns with ONT is that the read accuracy is low and this may lead to issues in assembly of complex polyploids with similar subgenomes. I suspect that based on this study, it is clear that this is a valid concern for some assemblers, but may have been overcome in others. Though none of this is explored or discussed. Again, is there any information in the literature contrasting assemblers that could provide insights into what you observed?
Searches at 90% identify and coverage for genes and TEs is not strict, especially with genomes that have highly identical subgenomes. If you reduce your thresholds enough, all features will map to your genome…..
The choice of language is often objective or not representative of the results. For example, the 'extremely' similar TE content between Renan and CS. Why not say it is similar and actually report a value or a % difference. This would be more concise and informative than using vague and overzealous language.
Page 8, short reads (dash or no dash?)
The font sizes in Figure 2 are very small.
The RNAseq is not really presented at all, except in the Materials and Methods. I thought the genes were ab initio predicted until I saw RNAseq in the materials and methods. I suggest at least making a note of RNAseq into the results and/or discussion since this additional effort does bring added value to the annotations and the manuscript. The discussion says de novo annotations, but I suggest explicitly stating that RNAseq was performed.
Figure 3 C and D do not have horizontal axis labels, the top should be labelled as subgenome, bottom as chromosome, and the vertical axis (not the top) should be labelled as number of gaps and chromosome length. Same comment for labelling of vertical axis for panels A and B, horizontal axis should be labelled as genome assemblies, which are reflected in the pallet/legend. Note that many of the colours in this pallet are similar and difficult to differentiate, it may actually take less space to label the bars with each wheat line to make it less cryptic.
How were the dotplots in figure 4 generated? Perhaps I missed it in the materials and methods. Also one of the axis have labels or units, etc.

Much of the text in Figure 5 is too small and illegible.
Page 10: The discussion is superficial and vague and should provide an accurate and pragmatic discussion of the results in the context of the literature. For example, the manuscript boasts a 'higher resolution'… but of what? Perhaps 'complex repetitive regions'? To reiterate my previous comment on the lack of literature support throughout the manuscript - Were these 'higher resolutions' of <complex repetitive regions> comparable to what was observed in the literature when ONT was applied to other systems? Again, these advantages of ONT and the assembly could be more thoroughly explored.

Methods

Are the methods appropriate to the aims of the study, are they well described, and are necessary controls included? Choose an item.

Conclusions

Are the conclusions adequately supported by the data shown? Choose an item.

Reporting Standards

Does the manuscript adhere to the journal's guidelines on minimum standards of reporting? Choose an item.

Choose an item.

Statistics

Are you able to assess all statistics in the manuscript, including the appropriateness of statistical tests used? Choose an item.

Quality of Written English

Please indicate the quality of language in the manuscript: Choose an item.

Declaration of Competing Interests

Please complete a declaration of competing interests, considering the following questions:

- Have you in the past five years received reimbursements, fees, funding, or salary from an organisation that may in any way gain or lose financially from the publication of this manuscript, either now or in the future?
- Do you hold any stocks or shares in an organisation that may in any way gain or lose financially from the publication of this manuscript, either now or in the future?
- Do you hold or are you currently applying for any patents relating to the content of the manuscript?

- Have you received reimbursements, fees, funding, or salary from an organization that holds or has applied for patents relating to the content of the manuscript?
- Do you have any other financial competing interests?
- Do you have any non-financial competing interests in relation to this paper?

If you can answer no to all of the above, write 'I declare that I have no competing interests' below. If your reply is yes to any, please give details below.

'I declare that I have no competing interests'

I agree to the open peer review policy of the journal. I understand that my name will be included on my report to the authors and, if the manuscript is accepted for publication, my named report including any attachments I upload will be posted on the website along with the authors' responses. I agree for my report to be made available under an Open Access Creative Commons CC-BY license (http://creativecommons.org/licenses/by/4.0/). I understand that any comments which I do not wish to be included in my named report can be included as confidential comments to the editors, which will not be published.

Choose an item.

To further support our reviewers, we have joined with Publons, where you can gain additional credit to further highlight your hard work (see: https://publons.com/journal/530/gigascience). On publication of this paper, your review will be automatically added to Publons, you can then choose whether or not to claim your Publons credit. I understand this statement.

Yes Choose an item.
