# Supplementary material for: Long-read and chromosome-scale assembly of the hexaploid wheat genome achieves high resolution for research and breeding
Source: Gigascience. 2022 Apr 28;11:giac034. doi: 10.1093/gigascience/giac034 (PMC9049114; doi:10.1093/gigascience/giac034)
Supplement: giac034_Reviewer_2_Report_Original_Submission — Gabriel Keeble-Gagnere -- 10/26/2021 Reviewed [file giac034_reviewer_2_report_original_submission.pdf]

Reviewer Report

Title: Long-read and chromosome-scale assembly of the hexaploid wheat genome achieves high resolution for research and breeding

Version: Original Submission **Date:** 10/26/2021

Reviewer name: Gabriel Keeble-Gagnere

Reviewer Comments to Author:

The authors report on a new assembly of a French wheat variety, Renan, using Oxford Nanopore sequencing technology combined with short read polishing, Bionano optical maps and Hi-C to validate chromosome-level ordering after anchoring to IWGSC RefSeq v2.1. This is the first study I know of to use Oxford Nanopore to assemble a complete wheat genome, and the results demonstrate that this technology (together with short read polishing, Bionano, Hi-C, etc) can be successfully applied to such a complex genome. Evidence is presented to support the quality of the assembly, but it is mostly at the global statistics level (eg: contig N50, total size of gaps) or macro-scale (whole chromosome dotplots). One detailed comparison between Renan and Chinese Spring of a biologically important region is presented.

The assembly is clearly of a high standard and is a valuable addition to the growing set of wheat varieties assembled to chromosome-scale. However, given the high quality of the IWGSC RefSeq v2.1 assembly (Zhu et al. (2021)), the claim that this assembly "achieves higher resolution for research and breeding" is quite strong and needs to be supported by more evidence. Given what is presented here, a more accurate statement might be "achieves higher contiguity and local completeness".

The high contig N50 of 2.2Mb is highlighted but I feel that more work is needed to demonstrate that the sequence is free of artefacts. The authors show in Figure 2 that this assembly has the lowest (though only slightly) complete BUSCO score out of the wheat genomes they compare with. Is it possible that some regions cause problems for the Oxford Nanopore technology and are either fragmented or completely absent from the assembly? Bionano maps were used but no evidence is presented to show the level of agreement with the assembled sequence and Bionano maps, as is done in Zhu et al. (2021).

In summary I think there are two key things to address:

1) More evidence supporting that the assembly is locally accurate, especially validation with alignment to Bionano maps;

2) Some results presented to relate this assembly to the existing chromosome-scale assemblies of wheat genomes.

To address these points, I think the following would greatly enhance the paper:

a) Using any method (eg: the method in Brinton et al. (2020)), identify identical-by-state haplotypes between Renan and Chinese Spring and the chromosome-scale assemblies from Walkowiak et al. (2020). This analysis would essentially produce a table which would be valuable supplementary data. A figure similar to Figure 3 (b) from Walkowiak et al. (2020) for a single chromosome, showing the regions of the existing wheat genomes sharing haplotypes with Renan would help place this genome into context.

b) This then defines large regions of the Renan assembly that can be directly compared at the base level

to other assemblies. Select 2 or 3 examples to show how the Renan sequence compares to the equivalent region in other assemblies, and show the Bionano validation of Renan sequence together with presence of genes and gaps in each assembly being compared. Since the sequences being compared here should be the same (based on the previous step above), the genes from the Renan annotation can be mapped across and directly compared. This would provide direct evidence for the higher quality assembly being claimed.
Figure 5 is a good comparison of a biologically important region, but it is unclear if the region in Chinese Spring and Renan is the same haplotype or not. This needs to be clarified at the start of this section. If the same, then the comparison is of two regions expected to be basically identical (and could be one of the examples used in the proposed comparison analysis above); if different, then that needs to frame the discussion since the region in Chinese Spring could theoretically contain different genes or more repeats, for example.
Centromeres are not mentioned, though it is known to be a particularly difficult region in wheat genome assemblies. How do the centromeres look in this assembly and how do they compare to previous wheat assemblies? Do the Bionano maps validate the assembly in the centromere region? The analysis in point a) above would identify centromeres in common with other assemblies.
Likewise, the distal ends of chromosome arms, including the telomere sequences, are known to cause problems for Hi-C ordering and orientation. Again, the Bionano alignments demonstrating correct ordering would be particularly valuable.
Figure 2 should be a supplementary figure.

Methods

Are the methods appropriate to the aims of the study, are they well described, and are necessary controls included? Choose an item.

Conclusions

Are the conclusions adequately supported by the data shown? Choose an item.

Reporting Standards

Does the manuscript adhere to the journal’s guidelines on minimum standards of reporting? Choose an item.

Choose an item.

Statistics

Are you able to assess all statistics in the manuscript, including the appropriateness of statistical tests used? Choose an item.

Quality of Written English

Please indicate the quality of language in the manuscript: Choose an item.

Declaration of Competing Interests

Please complete a declaration of competing interests, considering the following questions:

- Have you in the past five years received reimbursements, fees, funding, or salary from an organisation that may in any way gain or lose financially from the publication of this manuscript, either now or in the future?
- Do you hold any stocks or shares in an organisation that may in any way gain or lose financially from the publication of this manuscript, either now or in the future?
- Do you hold or are you currently applying for any patents relating to the content of the manuscript?
- Have you received reimbursements, fees, funding, or salary from an organization that holds or has applied for patents relating to the content of the manuscript?
- Do you have any other financial competing interests?
- Do you have any non-financial competing interests in relation to this paper?

If you can answer no to all of the above, write 'I declare that I have no competing interests' below. If your reply is yes to any, please give details below.

I declare that I have no competing interests

I agree to the open peer review policy of the journal. I understand that my name will be included on my report to the authors and, if the manuscript is accepted for publication, my named report including any attachments I upload will be posted on the website along with the authors' responses. I agree for my report to be made available under an Open Access Creative Commons CC-BY license (http://creativecommons.org/licenses/by/4.0/). I understand that any comments which I do not wish to be included in my named report can be included as confidential comments to the editors, which will not be published.

Choose an item.

To further support our reviewers, we have joined with Publons, where you can gain additional credit to further highlight your hard work (see: https://publons.com/journal/530/gigascience). On publication of this paper, your review will be automatically added to Publons, you can then choose whether or not to claim your Publons credit. I understand this statement.

Yes Choose an item.
