# Supplementary material for: Long-read and chromosome-scale assembly of the hexaploid wheat genome achieves high resolution for research and breeding
Source: Gigascience. 2022 Apr 28;11:giac034. doi: 10.1093/gigascience/giac034 (PMC9049114; doi:10.1093/gigascience/giac034)
Supplement: giac034_Reviewer_2_Report_Revision_1 — Gabriel Keeble-Gagnere -- 1/18/2022 Reviewed [file giac034_reviewer_2_report_revision_1.pdf]

Reviewer Report

Title: Long-read and chromosome-scale assembly of the hexaploid wheat genome achieves high resolution for research and breeding

Version: Revision 1 **Date:** 1/18/2022

Reviewer name: Gabriel Keeble-Gagnere

Reviewer Comments to Author:

Firstly, the revisions to the manuscript are a great improvement and help contextualise the assembly with other wheat genomes already published. The haplotype analysis allows identification of regions shared with other published wheat genomes to inform the comparisons presented.
The change in the title to "high resolution" is appropriate and more accurately reflects the results. I do wonder if Oxford Nanopore should be mentioned in the title since one of the main contributions of this article is to show the technology is capable of assembling the wheat genome to a high standard.
The main remaining concern I have is around the claim that this assembly provides the "most contiguous and complete" (stated in the abstract) assembly of bread wheat to date.
As Reviewer 1 also noted, what is needed is a consideration of the pros and cons of this ONT-based assembly compared to other approaches that have been published before. It is clear that this method is capable of producing a high quality assembly, but given the already high quality of wheat assemblies, what exactly does this approach do better and what are its limitations? Is higher contiguity with lower base-level accuracy preferable to high base-level accuracy with lower local contiguity (small gaps)?
In this context the following points aim to highlight some remaining issues with suggestions on how to address them - in each case the aim is to be able to make a definite statement that informs the reader about the relative merits of this assembly.
- The authors have added a useful "Base accuracy" metric to Table 1, which shows this assembly to score lower than IWGSC RefSeq v2.1 (which is mostly short-read based, with some areas replaced with Pacbio sequence). At the same time, if the lower quality is mainly due to problems in repeat regions, this may be acceptable to some users of the sequence, provided the gene space is highly accurate. The authors note (line 168) that "coding regions… may have higher precision." I feel this needs to be explored - one way to do this might be to also include the base accuracy analysis only on gene space (say including 5kb around the gene). This would enable a concrete statement to be made about the overall gene space accuracy.
- The Bionano alignment shown in Figure 7d looks to me to show some ambiguity, with evidence of a collapsed and/or missing region, precisely within the dense cluster of genes. Without extra information here, given the Bionano alignment, it is hard to be certain that some genes have not been missed or that some genes could be collapsed/chimeric. Could RNA-seq alignments to the assembly be used to validate the gene models here? Or, given the size of the ONT reads, a small sub-region including a few genes could be shown together with the long and short reads aligning across it (presumably a single long read could include full length genes - which would be good evidence to back up the accuracy claims and show the value of long reads). This would enable a concrete statement to be made about the accuracy of

these gene models in Renan.
- Figure 8b aims to show the higher contiguity of the Renan assembly vs the equivalent region in Landmark. We can clearly see Renan has fewer gaps, but it's also clear that large regions, which are present in Landmark, are completely absent from the Renan assembly. The only comment about this is on lines 359-360 but given the claims of being "more complete", I feel it needs to be addressed more thoroughly. For example, mapping the genes in this region from Landmark to Renan, and the genes from Renan to Landmark (it has to be done both ways to avoid biases in the annotation method), and detailing any present in one and absent from the other, would at least give some biological context to consider, and enable a concrete statement to be made about coverage of the gene space in the two assemblies in this region. As it is presented, I find this example undermines the claim of completeness.
A few further points:
- Figure 7 needs the haplotype context around this region as shown in Figures 4, 5, 6 and 8. I am guessing the haplotype in this region is different between Renan and Chinese Spring, in which case the text around this figure should be clearer. As I read it I'm not sure if an identical region is being compared (in which case the two assemblies can be directly compared and conclusions made about their pros/cons) or different (in which case interpretation of the two assemblies is not as simple). If different, then it should be made clear that eg: 7b highlights the structural differences between haplotypes (to avoid potentially interpreting it as assembly inconsistencies). Also, the value of 7c is unclear since it is not surprising genes from Renan map well to itself and poorly to genes we already know have gaps. The legend for 7c should also make clear that it is the Renan genes being mapped to both regions (or if I've misunderstood, please clarify).
- Figure 8 describes the Sm1 locus and notes that Landmark and Renan are known carriers of the gene. But the haplotypes shown in 8a, in the roughly 1Mb around the gene, look quite different in the figure. The text says: "This analysis revealed a strong similarity of the Sm1 locus between CDC Landmark and Renan (Figure 8a), the presence of the Sm1 gene in blocks shared between the two cultivars." ("including" should be added after the comma) (lines 355-256) How to interpret this? Is the Sm1 gene and surrounding sequence exactly the same in Landmark and Renan? If so, this should be shown/clarified and the apparent difference in haplotypes shown in 8a explained.
- Figure 6a is a little confusing as it shows Ae. ventricosa reads covering most of 2D, but is presumably background noise, while the introgression has zero coverage. Could this be explained more clearly? Similarly for part b, the introgression has similar coverage to other parts of the chromosome which are not from Ae. ventricosa. Do the alignments need to be filtered more strictly?
- Figure 6b, while interesting, is a distraction as it does not relate to Renan. I would argue to only include parts a and c, since they are good examples of a candidate introgression shared between Renan and other varieties (part a) and something unique to Renan (part c).
- The section starting on line 325: All references to Figure 5 should be Figure 7.
- The labels on Figure 5d don't make sense (eg: 32.5M appears twice).
- Figure S1 needs scales added.
- Lines 322-324 and Figure S4 are not new information (reported in Walkowiak et al., 2020) and do not relate to Renan so I think they are a distraction.

Methods

Are the methods appropriate to the aims of the study, are they well described, and are necessary controls included? Choose an item.

Conclusions

Are the conclusions adequately supported by the data shown? Choose an item.

Reporting Standards

Does the manuscript adhere to the journal's guidelines on minimum standards of reporting? Choose an item.

Choose an item.

Statistics

Are you able to assess all statistics in the manuscript, including the appropriateness of statistical tests used? Choose an item.

Quality of Written English

Please indicate the quality of language in the manuscript: Choose an item.

Declaration of Competing Interests

Please complete a declaration of competing interests, considering the following questions:

- Have you in the past five years received reimbursements, fees, funding, or salary from an organisation that may in any way gain or lose financially from the publication of this manuscript, either now or in the future?
- Do you hold any stocks or shares in an organisation that may in any way gain or lose financially from the publication of this manuscript, either now or in the future?
- Do you hold or are you currently applying for any patents relating to the content of the manuscript?
- Have you received reimbursements, fees, funding, or salary from an organization that holds or has applied for patents relating to the content of the manuscript?
- Do you have any other financial competing interests?
- Do you have any non-financial competing interests in relation to this paper?

If you can answer no to all of the above, write 'I declare that I have no competing interests' below. If your reply is yes to any, please give details below.

I declare that I have no competing interests.

I agree to the open peer review policy of the journal. I understand that my name will be included on my report to the authors and, if the manuscript is accepted for publication, my named report including any attachments I upload will be posted on the website along with the authors' responses. I agree for my report to be made available under an Open Access Creative Commons CC-BY license (http://creativecommons.org/licenses/by/4.0/). I understand that any comments which I do not wish to be included in my named report can be included as confidential comments to the editors, which will not be published.

Choose an item.

To further support our reviewers, we have joined with Publons, where you can gain additional credit to further highlight your hard work (see: https://publons.com/journal/530/gigascience). On publication of this paper, your review will be automatically added to Publons, you can then choose whether or not to claim your Publons credit. I understand this statement.

Yes Choose an item.
