# Supplementary material for: Long-read and chromosome-scale assembly of the hexaploid wheat genome achieves high resolution for research and breeding
Source: Gigascience. 2022 Apr 28;11:giac034. doi: 10.1093/gigascience/giac034 (PMC9049114; doi:10.1093/gigascience/giac034)
Supplement: giac034_Reviewer_2_Report_Revision_2 — Gabriel Keeble-Gagnere -- 2/21/2022 Reviewed [file giac034_reviewer_2_report_revision_2.pdf]

Reviewer Report

Title: Long-read and chromosome-scale assembly of the hexaploid wheat genome achieves high resolution for research and breeding

Version: Revision 2 **Date:** 2/21/2022

Reviewer name: Gabriel Keeble-Gagnere

Reviewer Comments to Author:

Thanks to the authors for these revisions. I am satisfied with the responses to the points raised and now recommend this manuscript for publication. This resource is a great addition to the growing number of chromosome-scale wheat genome assemblies.
A couple of minor points:
- With regards the analysis presented in Figure 7: I understand now that the complete Chinese Spring protein sequences coming from Huo et al. (2018) were annotated against the Pacbio-based Zimin et al. (2017) assembly. This explains why they are more complete compared to the IWGSC assembly being compared here. To make it absolutely clear, I suggest wording lines 644-646 something like this: "Protein sequences of prolamin and resistance genes[35] annotated against the Pacbio-based assembly of Chinese Spring[5], were downloaded and aligned to the IWGSC RefSeq v2.1 and Renan assemblies using BLAT[48] with default parameters."
- The authors only mention "IWGSC RefSeq v2.1" once in the text (line 589) - it is usually referred to as "CS RefSeq v2.1" (sometimes "Chinese Spring RefSeq v2.1") in the text or "CS" in legends and figures. I wonder if it should be explicitly referred to as IWGSC RefSeq v2.1 throughout or at least made clear somewhere that CS = IWGSC RefSeq v2.1. Either way it should be consistent.

Methods

Are the methods appropriate to the aims of the study, are they well described, and are necessary controls included? Choose an item.

Conclusions

Are the conclusions adequately supported by the data shown? Choose an item.

Reporting Standards

Does the manuscript adhere to the journal's guidelines on minimum standards of reporting? Choose an item.

Choose an item.

Statistics

Are you able to assess all statistics in the manuscript, including the appropriateness of statistical tests used? Choose an item.

Quality of Written English

Please indicate the quality of language in the manuscript: Choose an item.

Declaration of Competing Interests

Please complete a declaration of competing interests, considering the following questions:

- Have you in the past five years received reimbursements, fees, funding, or salary from an organisation that may in any way gain or lose financially from the publication of this manuscript, either now or in the future?
- Do you hold any stocks or shares in an organisation that may in any way gain or lose financially from the publication of this manuscript, either now or in the future?
- Do you hold or are you currently applying for any patents relating to the content of the manuscript?
- Have you received reimbursements, fees, funding, or salary from an organization that holds or has applied for patents relating to the content of the manuscript?
- Do you have any other financial competing interests?
- Do you have any non-financial competing interests in relation to this paper?

If you can answer no to all of the above, write 'I declare that I have no competing interests' below. If your reply is yes to any, please give details below.

I declare that I have no competing interests.

I agree to the open peer review policy of the journal. I understand that my name will be included on my report to the authors and, if the manuscript is accepted for publication, my named report including any attachments I upload will be posted on the website along with the authors' responses. I agree for my report to be made available under an Open Access Creative Commons CC-BY license (http://creativecommons.org/licenses/by/4.0/). I understand that any comments which I do not wish to be included in my named report can be included as confidential comments to the editors, which will not be published.

Choose an item.

To further support our reviewers, we have joined with Publons, where you can gain additional credit to further highlight your hard work (see: https://publons.com/journal/530/gigascience). On publication of this paper, your review will be automatically added to Publons, you can then choose whether or not to claim your Publons credit. I understand this statement.

Yes Choose an item.
